# Glycolysis and T cell-associated gene signature predicts prognosis and therapeutic responses in pancreatic cancer

**DOI:** 10.3389/fimmu.2026.1918556

**Published:** 2026-08-28

**Authors:** Wancheng Li, Dongao Fan, Yan Du, Lin Li, Wenjia Li, Wence Zhou

**Affiliations:** 1 The Second School of Clinical Medicine, Lanzhou University, Lanzhou, China; 2 Department of General Surgery, Lanzhou University Second Hospital, Lanzhou, China; 3 Department of Medical Insurance Office, The Gansu Provincial Maternity and Child-Care Hospital, Lanzhou, China; 4 Department of Anesthesiology, Lanzhou University Second Hospital, Lanzhou, China

**Keywords:** drug sensitivity, glycolysis, GPR87, pancreatic cancer, prognosis, T cell

## Abstract

**Background:**

Pancreatic cancer (PC) is a fatal malignancy, with glycolysis and T cells playing crucial roles in its pathogenesis. This study explored prognosis-related genes in glycolysis and T cells in PC using bioinformatics methods.

**Methods:**

We first quantified distinct T-cell subsets via immune infiltration analysis of the TCGA-PAAD cohort and extracted T cell-related genes (T-RGs). Differentially expressed genes (DEGs) were obtained from GSE28735 and TCGA-PAAD. Intersecting these DEGs with T-RGs and glycolysis-related genes (G-RGs) yielded candidate genes. We screened prognostic genes using univariate Cox and LASSO regression, built a prognostic model in TCGA-PAAD and validated it in GSE57495. A nomogram was constructed for survival prediction, its reliability verified by calibration and ROC curves. We compared immune microenvironment, pathway enrichment, mutation landscape and drug sensitivity between high- and low-risk subgroups, and built lncRNA-miRNA-mRNA regulatory networks. *In vitro* and *in vivo* mouse tumorigenesis assays explored GPR87’s potential involvement in glycolytic activity and CD8^+^ T cell infiltration. Seahorse analysis, glucose uptake detection and immunohistochemistry further explored roles in glucose metabolism and anti-tumor immunity.

**Results:**

*MET*, *KDELR3*, *AK4*, and *GPR87* were determined as prognostic genes. In the training set, this exploratory prognostic model showed moderate predictive performance, with area under the curve values of 0.72, 0.71, and 0.71 at 1, 2, and 3 years, respectively. The risk score and N-stage were identified as independent prognosis predictors, and the developed nomogram demonstrated moderate predictive performance. Functional pathways revealed enrichment in 44 pathways. There were 17 differential immune cells. In addition, risk scores were correlated with 28 immune checkpoints. The regulatory network comprised 26 miRNAs and 50 lncRNAs. Furthermore, computationally predicted correlations between prognostic genes and 60 drugs were identified in different risk groups. *In vitro* and *in vivo* experiments demonstrated that *GPR87* knockdown inhibits the proliferation, migration, and clonogenic ability of PC cells while promoting their apoptosis. Furthermore, *GPR87* expression was negatively correlated with CD8^+^ T cell, and *GPR87* knockdown inhibited glycolysis in pancreatic cancer cells.

**Conclusion:**

*MET*, *KDELR3*, *AK4*, and *GPR87* were identified as candidate prognostic genes in PC, providing preliminary hypothesis-generating insights that require validation in independent institutional or prospective clinical cohorts before any clinical application.

## 1 Introduction

Pancreatic cancer (PC) is a highly aggressive malignancy of the digestive system. Due to its subtle early clinical manifestations, as well as the absence of effective screening methods, most patients are only diagnosed at intermediate or advanced stages, resulting in the loss of opportunities for radical resection (1, 2). Only approximately 20% of patients are eligible for surgical resection at diagnosis, and even after surgery, recurrence is common, contributing to a 5-year survival rate below 13%. Therefore, there is an urgent need to identify novel prognostic biomarkers and therapeutic strategies to enhance the clinical management of PC.

In recent years, tumor immunotherapy has achieved remarkable success in multiple malignancies; however, its clinical efficacy in PC remains limited (3). This poor therapeutic efficacy is largely attributed to the unique immunosuppressive tumor microenvironment (TME) of PC (4). In the PC TME, cytotoxic CD8^+^ T cells, which represent the core effector cells of antitumor immunity, are frequently infiltrated but predominantly exist in a state of functional exhaustion or dysfunction, with severely impaired proliferation, cytokine production, and cytotoxic capacity (5, 6). Thus, investigating the mechanisms underlying T cell dysfunction is critical to overcoming immunotherapy resistance and improving the prognosis of patients with PC.

Metabolic reprogramming is a hallmark of PC (7). Notably, aberrantly elevated aerobic glycolysis (the Warburg effect), driven by oncogenic mutations such as KRAS, provides energy and biosynthetic precursors for rapid tumor proliferation and profoundly shapes the immunosuppressive TME (8). By competing for glucose and secreting lactate, tumor cells create a nutrient-depleted, acidic milieu that suppresses T cell activation, impairs effector function, and promotes the expansion of immunosuppressive subsets such as regulatory T cells (9). These findings indicate a close and dynamic crosstalk between tumor glycolytic metabolism and T cell immune function. Therefore, identifying prognostic biomarkers associated with this immunometabolic regulation would aid in elucidating the mechanisms of immune cell dysfunction and provide novel targets for personalized therapy (10).

Currently, bioinformatics approaches are widely used to identify novel biomarkers and potentially effective small-molecule drugs in PC (10, 11). In this study, we aimed to identify candidate genes associated with glycolysis-related genes (G-RGs) and T cell-related genes (T-RGs) in patients with PC via transcriptomic analysis. Univariate Cox regression and least absolute shrinkage and selection operator (LASSO) regression were applied to screen prognostic genes and establish a prognostic model for PC. We further analyzed the enriched pathways, immune profiles, and TME characteristics of patients with PC stratified by the risk score. Finally, *in vitro* and *in vivo* experiments preliminarily verified the effects of GPR87, a gene included in the risk score, on the malignant biological behaviors of PC.

## 2 Materials and methods

### 2.1 Data source

Transcriptomic data from patients with PC and normal pancreatic tissue samples were obtained from the Gene Expression Omnibus (GEO) (https://www.ncbi.nlm.nih.gov/geo/) and The Cancer Genome Atlas (TCGA) (https://gdc.cancer.gov/). The GSE28735 dataset (45 PC samples and 45 normal samples) served as the primary dataset for differential expression analysis (12, 13). TCGA-PAAD (177 PC samples with survival information and 4 normal samples) was used as the training cohort (14). Three independent GEO datasets were used for validation: GSE62452 (65 PC cases with complete survival data) (15), GSE57495 (63 PC samples with survival data) (16), and GSE79668 (49 PC samples with survival information (17). Samples with missing survival time, survival status, or key clinical variables were excluded prior to analysis. For TCGA-PAAD, differential expression analysis was performed on count data using DESeq2, whereas all downstream analyses (including GSEA, GSVA, LASSO modeling, and survival analysis) used FPKM-normalized expression data. A total of 296 glycolysis-related genes (G-RGs) were retrieved from the Molecular Signatures Database (MSigDB v7.5.1) (https://www.gsea-msigdb.org/gsea/msigdb/) after removing duplicates (18). Each dataset was independently analyzed without cross-platform integration or batch correction. Detailed data download and preprocessing procedures are provided in the **Supplementary Methods**.

### 2.2 Identification of T-RGs

The single-sample gene set enrichment analysis (ssGSEA) algorithm was applied to TCGA-PAAD to compute enrichment scores for 28 immune cell types across PC and normal samples using the R package GSVA (19). Immune cells showing significant abundance differences between the two groups were identified as differential immune cells (Wilcoxon test, p < 0.05). Among the T cell-related differential immune cells, those whose high/low ssGSEA score groups showed significantly different overall survival (Kaplan-Meier (KM) analysis, p < 0.05) were selected as traits for downstream co-expression analysis (20). Weighted Gene Co-expression Network Analysis (WGCNA) was subsequently performed on TCGA-PAAD to identify gene modules correlated with the selected T cell ssGSEA scores (21) After outlier removal and soft-threshold selection to satisfy scale-free network topology (R² ≥ 0.90), co-expression modules were constructed using a dynamic tree-cutting algorithm. Modules showing a significant correlation with T cell ssGSEA scores (|cor| > 0.3 and p < 0.05) were designated as T cell-related gene sets and defined as T-RGs. Detailed WGCNA parameter settings are provided in the **Supplementary Methods**.

### 2.3 Identification of candidate genes and functional analysis

Differentially expressed genes (DEGs) were identified in GSE28735 (DEGs1) and TCGA-PAAD (DEGs2) using limma (v 3.54.0) and DESeq2 (v 1.38.0) (22), respectively, with a threshold of |log2 Fold Change (FC)| > 0.5 and p < 0.05.The threshold were selected to balance sensitivity and biological relevance. The candidate genes were subsequently refined through dataset intersection (GSE28735 and TCGA-PAAD), overlap with T-RGs/G-RGs, Cox regression, and LASSO analysis to improve robustness (23). DEGs were identified using DESeq2 (v1.38.0) in TCGA-PAAD with the same criteria. Volcano plots and heatmaps were generated using ggplot2 and ComplexHeatmap (v2.14.0) (24). Differentially expressed genes from GSE28735 and TCGA-PAAD were intersected using VennDiagram (v1.7.3) (25), generating DEGs3. Candidate genes were obtained by overlapping DEGs3 with T-RGs and G-RGs. Gene Ontology (GO) and Kyoto Encyclopedia of Genes and Genomes (KEGG) enrichment analyses were conducted using “clusterProfiler” (v 4.7.1.003) (26) and visualized with “ggpubr” (v 0.6.0) (27) (p-adjust < 0.05). GeneMANIA was used to predict functional associations.

### 2.4 Screening of prognostic genes and constructing a prognostic model

Univariate Cox regression was performed on candidate genes using TCGA-PAAD samples with available survival information (hazard ratio (HR) ≠ 1, p < 0.05) (28) This threshold was designed to retain sufficient candidate genes for subsequent least absolute shrinkage and selection operator (LASSO) analysis while controlling the false-positive rate. Genes passing the proportional hazards (PH) assumption test (p > 0.05) were retained as prognosis-related genes. LASSO regression was subsequently performed using “glmnet” (v 4.1-4) (29) with the optimal lambda value (lambda.min) determined via 10-fold cross-validation to identify prognostic genes and control for overfitting. The risk score was calculated as:


$$\text{risk score}=\sum_{\text{i}=1}^{\text{n}}(\text{coefi}*\text{Xi}),$$

where “coef” represents the risk coefficient for each prognostic gene, and “X” represents the expression level of that gene in the respective sample.

Expression differences in prognostic genes were further evaluated in the GSE28735 and GSE62452 datasets (Wilcoxon test, p < 0.05), and Kaplan-Meier (KM) survival analysis was performed to assess their prognostic significance. Detailed visualization methods are provided in the **Supplementary Methods**.

### 2.5 Assessment and verification of the prognostic model

The risk scores for PC in TCGA-PAAD, GSE57495 and GSE79668 were calculated. Due to inter-dataset heterogeneity, dataset-specific median risk scores were used for risk stratification rather than a unified cutoff. This strategy reduces bias from expression-scale differences and has been applied in previous prognostic studies (30). The generalizability of risk stratification was further evaluated in an independent cohort. KM survival analysis was performed for the two risk groups using “survival” (v 3.5-3) (31) (p < 0.05). Receiver operating characteristic (ROC) curves were generated at 1-, 2-, and 3-year time points, and the area under the curve (AUC) was calculated (AUC > 0.6) using “survivalROC” (v 1.0.3.1) (32). Heatmaps were plotted using “pheatmap.” In addition, the median risk score threshold from TCGA-PAAD was applied to the GSE79668 dataset for risk stratification and validation analysis.

### 2.6 Independent prognostic analysis

Clinical characteristics including risk score, age, gender, and T and N stage were assessed for compliance with the PH assumption prior to Cox regression (p > 0.05). Univariate Cox regression was performed in TCGA-PAAD to identify variables significantly associated with prognosis (HR ≠ 1, p < 0.05) using “survival” package (33). The independent prognostic factors were identified using a multivariable Cox regression model (HR ≠ 1 and p < 0.05). A nomogram was constructed using the R package “rms” (v 6.5.9) (34). Model performance was evaluated by ROC curves (AUC > 0.6), calibration curves at 1, 2, and 3 years, and decision curve analysis (DCA) to assess clinical net benefit. Risk score distributions across clinical subgroups were compared using the Wilcoxon test (p < 0.05).

### 2.7 Gene set enrichment analysis

In TCGA-PAAD, the two risk groups of patients with PC were analyzed using “DESeq2.” The log_2_FC values were sorted from largest to smallest, and then GSEA was predicted using the “clusterProfiler.” The c2.cp.kegg.v2023.1.Hs.symbols.gmt obtained from MSigDB (https://www.gsea-msigdb.org/gsea/msigdb) was used as the background gene set, and “clusterProfiler” was used to perform GSEA. The significance threshold for GSEA results was set at |normalized enrichment score (NES)| > 1 and p < 0.05. The significantly enriched pathways were visualized using the “enrichplot” (v 1.20.3) (35). The top five most significant pathways, based on p-values, were selected for visual presentation.

### 2.8 Immune microenvironment analysis

Stromal, immune, and ESTIMATE scores were computed for TCGA-PAAD PC samples using the ESTIMATE algorithm, and differences between risk groups were assessed by the Wilcoxon test (p < 0.05). The expression of 48 immune checkpoints was compared between risk groups. Violin plots were created using the “ggplot2” for visualization of the expression of 48 immune checkpoints (36). The correlations between prognostic genes (MET, KDELR3, AK4, and GPR87) and differential immune checkpoints were analyzed using Spearman’s correlation (|cor| > 0.3 and p < 0.05). Furthermore, box plots were created using “ggplot2” to visually demonstrate the differences in 28 immune cells (37) enrichment scores between the two risk groups. The ssGSEA was used to compute these levels. Immune infiltration scores of 28 immune cell types were visualized using pheatmap and compared between risk groups using the Wilcoxon rank-sum test (rstatix, p<0.05). Box plots were generated with ggplot2. Spearman analysis (psych; |cor|>0.3, p<0.05) was performed to evaluate correlations between immune cells and prognostic genes. TMB data were obtained from TCGA-PAAD. The R package “maftools” (v 2.18.0) (38) was used to analyze TMB in the two PC risk groups in TCGA-PAAD. Subsequently, an analysis of prognostic gene mutations was conducted using “maftools.

### 2.9 Regulatory networks

The microRNAs (miRNAs) targeting each prognostic gene were forecasted using the miRanda (https://regendbase.org/tools/miranda) and MicroRNA Target Prediction (miRDB) (https://mirdb.org/) databases with the R package “multiMiR” (v 1.24.0) (39). The miRNAs obtained from these two databases were intersected using “VennDiagram” to identify the common miRNAs. Subsequently, the long non-coding RNAs (lncRNAs) corresponding to these common miRNAs were forecasted using starBase (http://starbase.sysu.edu.cn/). Finally, the network was constructed using “ggraph” (v 2.2.1).

### 2.10 Drug sensitivity

In TCGA-PAAD, the “pRRophetic” (v 0.5) (40) was employed to determine the half-maximal inhibitory concentration (IC_50_) for drugs from Genomics of Drug Sensitivity in Cancer (https://www.cancerrxgene.org/). Subsequently, “psych” was used to perform Spearman correlation analyses. This analysis was conducted with the aim of screening drugs based on the strength of their correlation between the IC_50_ of the drugs used and the risk scores (|cor| > 0.3 and p < 0.05). The Wilcoxon test was applied to compute the disparities in IC_50_ for the top 15 drugs (ordered by p-value) between the two groups. For visualization, “ggplot2” was used (p < 0.05).

### 2.11 Immune cell infiltration algorithms

CD8^+^ T cell infiltration levels in the ICGC-AU, GSE183795, and GSE717295 cohorts were quantified using five independent immune deconvolution algorithms: CIBERSORT, EPIC, MCP-counter, quanTIseq, and xCell, integrated via the R package immunedeconv. The correlation between CD8^+^ T cell abundance estimates and GPR87 expression was evaluated across all three cohorts.

### 2.12 Cell and molecular biology analysis

#### 2.12.1 Cell lines, culture conditions, and cell transfection

Human PC cell lines BxPC-3 and PANC-1 (KRAS G12D mutation) (Cell Bank of the Chinese Academy of Sciences, Shanghai, China) and hTERT-HPNE cells (Cell Bank of the Chinese Academy of Sciences, Shanghai, China) were used. The BxPC-3 cells were cultured in RPMI-1640, whereas the PANC-1 and HPNE cells were maintained in DMEM, each supplemented with 10% fetal bovine serum (FBS) and 1% penicillin–streptomycin. Cells were incubated at 37 °C with 5% CO_2_. Small interfering RNAs (siRNAs) (TsingKe, Beijing, China) were transfected using Lipofectamine 3000 (Thermo Fisher Scientific) as per the manufacturer’s protocol. The siRNA transfection concentration was 50 nM. Cells were collected 24 h after transfection, and the interference efficiency was detected by reverse transcription quantitative PCR (RT-qPCR). Transfected cells in the logarithmic growth phase were digested and seeded for subsequent cell phenotypic experiments.

#### 2.12.2 RNA extraction and RT-qPCR

Total RNA was extracted with TRIzol (Vazyme), reverse-transcribed using HiScript Q RT SuperMix (Vazyme), and quantified by RT-qPCR with ChamQ SYBR qPCR Master Mix (Vazyme). Normalization was performed using β-actin as the reference gene. The primer and siRNA sequences are provided in **Supplementary Table 1**.

#### 2.12.3 Glucose uptake

Glucose uptake was detected using the 2-NBDG Glucose Uptake Assay Kit (Servicebio, G1744) following the manufacturer’s protocol. Briefly, after being seeded and attached, cells were starved in glucose-free and serum-free medium for 1 h. The diluted 2-NBDG working solution was then added to incubate the cells at 37 °C for 50 min before fluorescence microscopic observation. Fluorescence intensity of cells in each group was quantified using ImageJ software. Values were normalized to the control group to calculate the relative glucose uptake intensity.

#### 2.12.4 Extracellular acidification rate

ECAR was measured using the Seahorse XF Glycolysis Stress Test Kit (Cat. No. 103020-100, Agilent Technologies, USA) according to the manufacturer’s instructions. Briefly, cells were seeded in XF24 microplates (Cat. No. 103730-100, Agilent) and incubated overnight to allow attachment. Before the assay, cells were gently washed with phosphate-buffered saline (PBS) and then maintained in XF Assay Medium (Cat. No. 103680-100, Agilent) supplemented with 10 mM glucose, 2 mM L-glutamine, and 1 mM sodium pyruvate. The Seahorse XFe24 Extracellular Flux Analyzer (Agilent) automatically injected metabolic modulators at designated time points and continuously recorded ECAR in real time throughout the detection period.

### 2.13 Western blotting

Total cellular protein was extracted. Protein concentration was quantified using a BCA protein quantification kit (Solarbio, Cat: PC0020). Equal amounts of protein samples were separated via SDS-PAGE at an appropriate gel concentration. Separated proteins were transferred onto PVDF membranes (Solarbio, Cat: YA1701). Membranes were blocked and incubated with primary antibodies followed by secondary antibodies in sequence. Bands were visualized with ECL chemiluminescence reagent (EpiZyme, SQ201). The corresponding antibody dilutions were prepared according to the manufacturer’s instructions. Antibody information: α-Tubulin(proteintech, 66031-1-Ig) information:GPR87 (Immunoway, YT5438), HK2 (Boster, Clone#MA01389).

### 2.14 Animal models

Female BALB/c nude mice (4 weeks old; Cavens, Changzhou, China) were maintained under specific pathogen-free conditions. For xenografts, mice (n = 5/group) received subcutaneous injections of 1 × 10^6^ cells into the right lumbar region. After 4 weeks, the mice were euthanized, and tumors were collected for immunohistochemistry or stored at -80 °C.

Orthotopic pancreatic tumors were harvested from KPC mice (Beijing Vitalstar Biotechnology Co., Ltd) and minced into ~3 mm³ fragments. Tumor pieces were mixed with high-concentration basement membrane matrix (Matrigel, Corning, 354248) and injected subcutaneously into the right flank of 6-week-old female C57BL/6J mice to establish KPC allograft models. When allografts reached ~800 mm³, tumors were collected. Viable tumor regions (excluding necrotic cores) were selected and serially passaged into recipient mice using the same protocol. After 3 serial passages, once tumor growth kinetics stabilized, formal experiments were initiated. For third-passage tumor-bearing mice (n = 5/group), when tumors reached ~100 mm³, adeno-associated virus (AAV) vectors targeting GPR87 or negative control AAV (TsingKe, Beijing, China) were delivered via intratumoral injection, at a total dose of 5 × 10¹¹ viral genomes (v.g.) per mouse. The GPR87-targeting shRNA and negative control sequences are provided in **Supplementary Table 1**. Mice were euthanized when tumors reached ~1500 mm³ as the study endpoint. Subcutaneous allografts were harvested: portions of the tumor tissue were processed for immunohistochemistry (IHC), and the remaining samples were stored at -80 °C for later use.

Mice were placed in a sealed anesthesia chamber and anesthetized with 5% isoflurane (MCE, HY-A0134) delivered in pure oxygen at a flow rate of 1 L/min. After deep anesthesia was confirmed, the mice were rapidly euthanized by decapitation. All procedures were reviewed and approved by the Institutional Animal Care and Utilization Committee of the Second Hospital of Lanzhou University (approval number: D2025-786).

### 2.15 Immunohistochemical staining

Tumor tissues were fixed, paraffin-embedded, and sectioned at 4 μm for IHC staining. Primary antibodies against Ki-67 (Proteintech, 27309-1-AP), GPR87 (Immunoway, YT5438), CD8 (Proteintech, 66868-1-Ig, Servicebio, GB15068-50), LDHA (Boster, Clone#CFE-12) and c-MET(Proteintech, 25869-1-AP) were used. Quantification was performed using ImageJ by investigators blinded to group allocation, with five randomly selected fields analyzed per sample. Differences between groups were assessed using a two-tailed Mann–Whitney U test. Use of human tissue specimens was approved by the Ethics Committee of the Second Hospital of Lanzhou University (approval number: 2024A-1102).

### 2.16 Statistical analysis

Statistical analyses were performed using R software (v 3.9.1) and GraphPad Prism 8. The quantitative data are presented as the mean ± standard deviation. For *in vitro* experiments, comparisons between two groups were performed using a two-tailed Student’s t-test. For comparisons among multiple groups with one independent variable, one-way ANOVA was used, followed by Tukey’s multiple comparisons test. For experiments involving two independent variables, two-way ANOVA was performed, and interaction effects were assessed. Endpoint tumor volumes and tumor weights from mouse experiments were compared using a two-tailed Mann–Whitney U test. Unless otherwise stated, each experiment included at least three independent biological replicates. A p-value < 0.05 was considered statistically significant.

## 3 Results

### 3.1 T-RGs identified by WGCNA

**Figure 1:**
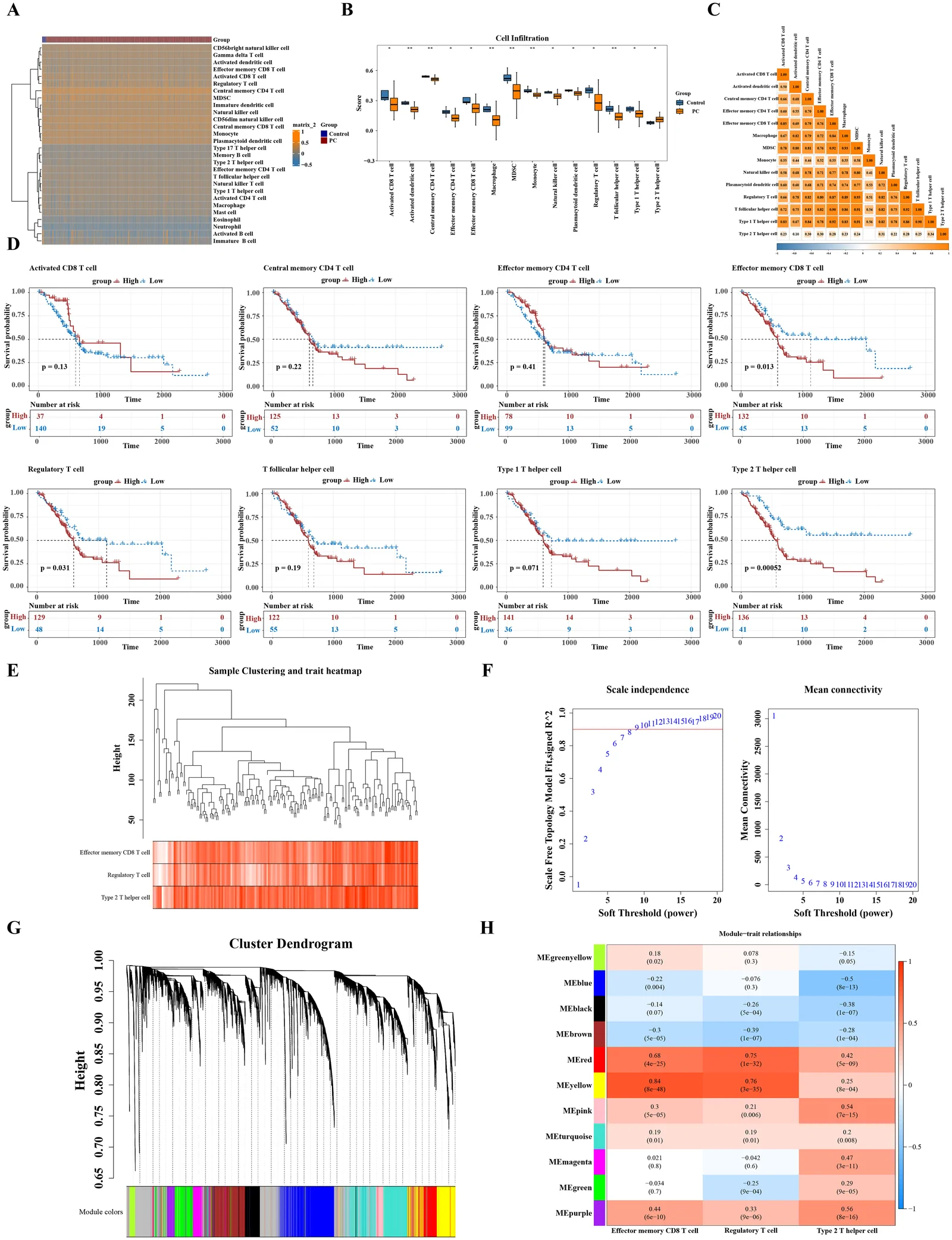
Identification of T cell-related genes **(A)** Heat map generated by ssGSEA illustrates the abundance of immune-infiltrating cells in pancreatic cancer (PC) and adjacent samples from TCGA-PAAD. **(B)** Fourteen immune cell types exhibiting significant differences in abundance between PC and adjacent samples were identified (p < 0.05). **(C)** Differential immune cell correlation heatmap. An empty cell indicates p > 0.05. **(D)** Kaplan-Meier survival curves for patients with PC stratified into high- and low-risk score groups based on differential T cell abundance. **(E)** Hierarchical clustering of patients with PC samples in the training set. Each branch in the clustering tree represents a sample, and the vertical axis represents the Euclidean distance of the sample’s expression levels. **(F)** Selection of the soft threshold. To determine the optimal soft threshold, refer to the image on the left, where the red line indicates the selected value of the scale-free fit index. The x-axis in both images represents the power of the weighting parameter. The y-axis in the left image represents the scale-free fit index, while the y-axis in the right image represents the mean of the adjacency functions for all genes in the corresponding gene module. **(G)** Identification of co-expression modules. **(H)** Module correlation heatmap displaying ssGSEA scores for three types of T cells. **p < 0.01, and *p < 0.05.

In TCGA-PAAD, the immune-infiltrating cell enrichment fraction was calculated for each sample, and the ssGSEA heatmap illustrated the abundance of 28 immune cells across the PC and control groups (**Figure 1A**). A total of 14 differential immune cells exhibiting statistically significant differences in abundance between the PC and control groups (p < 0.05) were selected (**Figure 1B**). The correlation among differential immune cells was subsequently analyzed, revealing predominantly positive correlations (**Figure 1C**). Notably, regulatory T cells (Tregs) and myeloid-derived suppressor cells exhibited the strongest positive correlation (cor = 0.928, p < 0.05) (**Supplementary Table 2**). To investigate the influence of various T cell abundances on the survival of patients with PC, individuals in the TCGA-PAAD training set were categorized into high- and low-score groups based on the eight distinct T cell abundances identified in the previous analysis. KM survival curve analysis was then performed. There existed notable differences in the survival probability between groups with high- and low-scores for three T cell types: effector memory CD8^+^ T cells, regulatory T cells, and type 2 T helper cells (p < 0.05) (**Figure 1D**). The samples were clustered to eliminate anomalies. WGCNA results showed no significant outliers; thus, all samples were retained for subsequent analysis (**Figure 1E**). The optimal soft threshold (power) was selected as nine (R^2^ = 0.9100) (**Figure 1F**). Altogether 11 modules were obtained by constructing the co-expression network (**Figure 1G**). Among these 11 modules (not including gray modules that cannot be clustered), the two modules with all three T cell types with absolute values > 0.3, where cor > 0.3 and p < 0.05. The MEred and MEpurple modules were designated key modules, from which 1752 genes were extracted and defined as T-cell-related signature genes (T-RGs) (**Figure 1H**, **Supplementary Table 3**).

### 3.2 Identification and functional enrichment of candidate genes

**Figure 2:**
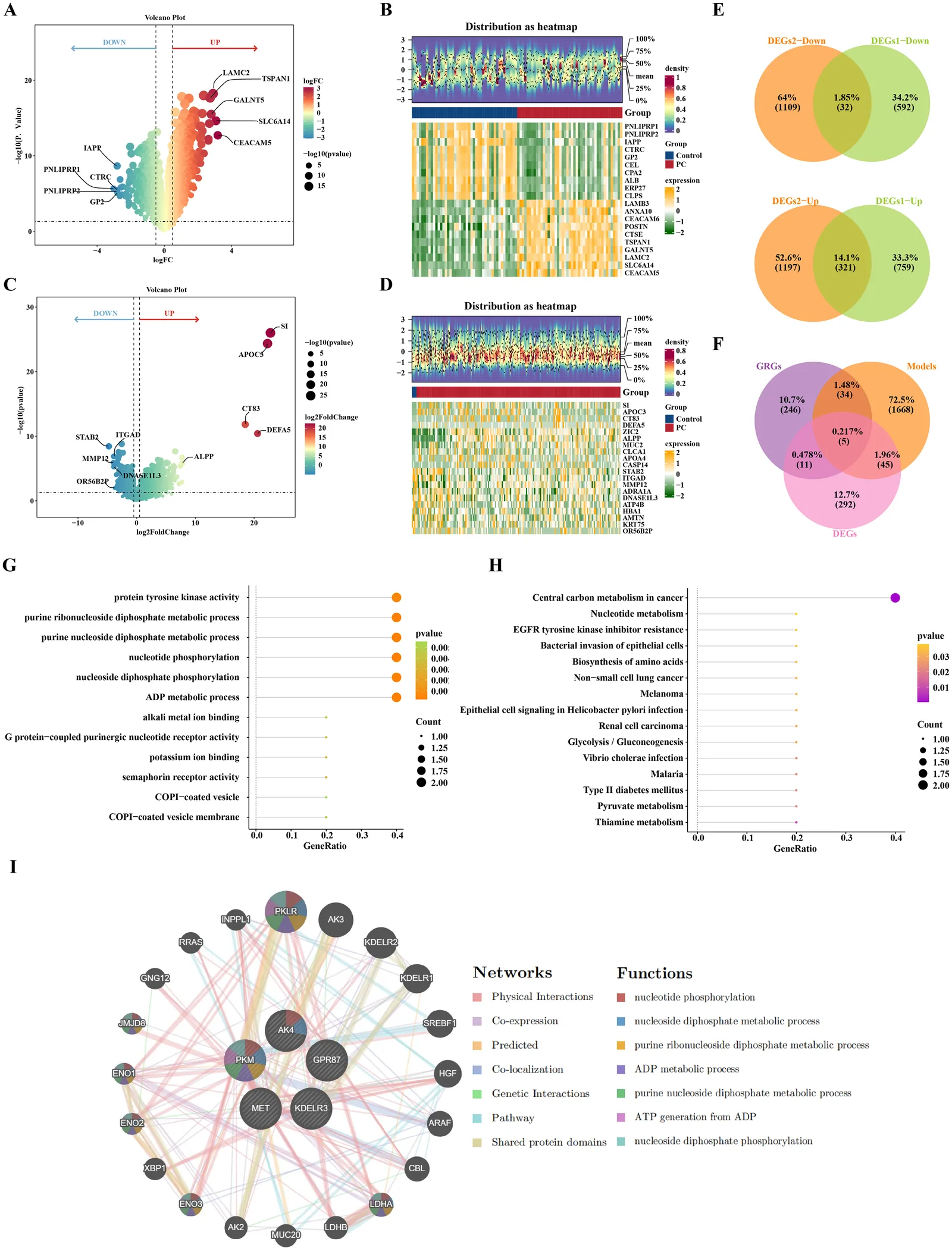
Identification and functional enrichment of candidate genes **(A)** Volcano plot illustrating differentially expressed genes in the GSE28735 dataset. **(B)** Heat map depicting differentially expressed genes in the GSE28735 dataset. **(C)** Volcano plot presenting differentially expressed genes in the TCGA-PAAD dataset. **(D)** Heat map showing differentially expressed genes in the TCGA-PAAD dataset. **(E)** Venn diagram showing the intersection of differentially expressed genes (DEGs3) from the GSE28735 and TCGA-PAAD datasets. **(F)** Venn diagram integrating the DEGs3, T-DEGs, and G-DEGs to identify five candidate genes. **(G)** Gene Ontology (GO) enrichment results for the five genes. The x-axis represents the proportion of genes mapped to each pathway relative to the total number of genes, the y-axis shows the names of the GO terms, the color gradient indicates the p-value, and the bubble size represents the number of genes contained in each GO term. **(H)** Kyoto Encyclopedia of Genes and Genomes (KEGG) enrichment results for the five genes. The x-axis represents the proportion of genes mapped to each pathway relative to the total number of genes, the y-axis shows the pathway names, the color gradient indicates the p-value, and the bubble size represents the number of genes contained in each pathway. **(I)** Twenty function-related genes were identified using GeneMANIA, leading to the construction of a gene–gene interaction network. The large circle in the middle represents the candidate gene, and the small circles on the outside represent genes related to the candidate gene.

A total of 1,704 DEGs1 were obtained in GSE28735, including 1,080 upregulated and 624 downregulated genes (**Figures 2A, B**). Meanwhile, 2,659 DEGs2 were screened in TCGA-PAAD; 1,518 were upregulated, and 1,141 were downregulated (**Figures 2C, D**). The intersection of the upregulated DEGs1 and upregulated DEGs2 yielded a total of 321 common upregulated genes. In addition, the intersection of the downregulated DEGs1 and downregulated DEGs2 resulted in 32 common downregulated genes. When combining these up- and downregulated intersecting genes, 353 DEGs3 were obtained (**Figure 2E**). By intersecting DEGs3, T-RGs, and G-RGs, a total of five candidate genes were identified (**Figure 2F**). A total of 108 GO biological functions, including 89 biological processes (BP), 2 cellular components (CC), and 17 molecular functions (MF) (**Figure 2G**; **Supplementary Table 4**), and 17 KEGG pathways were enriched for the candidate genes (**Figure 2H**; **Supplementary Table 5**). Functional enrichment showed that in BP, these candidate genes were primarily associated with ADP metabolic processes, nucleoside diphosphate phosphorylation, and nucleotide phosphorylation; in the CC, these candidate genes were primarily linked to COPI-coated vesicle membranes and COPI-coated vesicles; in the MF, these candidate genes were primarily involved in protein tyrosine kinase activity, semaphorin receptor activity, potassium ion binding, G protein-coupled purinergic nucleotide receptor and alkali metal ion binding. In terms of KEGG enrichment, 17 pathways were identified, including pyruvate metabolism and glycolysis/gluconeogenesis. Moreover, 20 genes with functions similar to those of the candidate genes were identified. The candidate genes were involved in a total of 51 functions. These functions were related to nucleotide phosphorylation and the metabolic process of nucleoside diphosphate (**Figure 2I**).

### 3.3 *MET*, *KDELR3*, *AK4* and *GPR87* as prognostic genes

**Figure 3:**
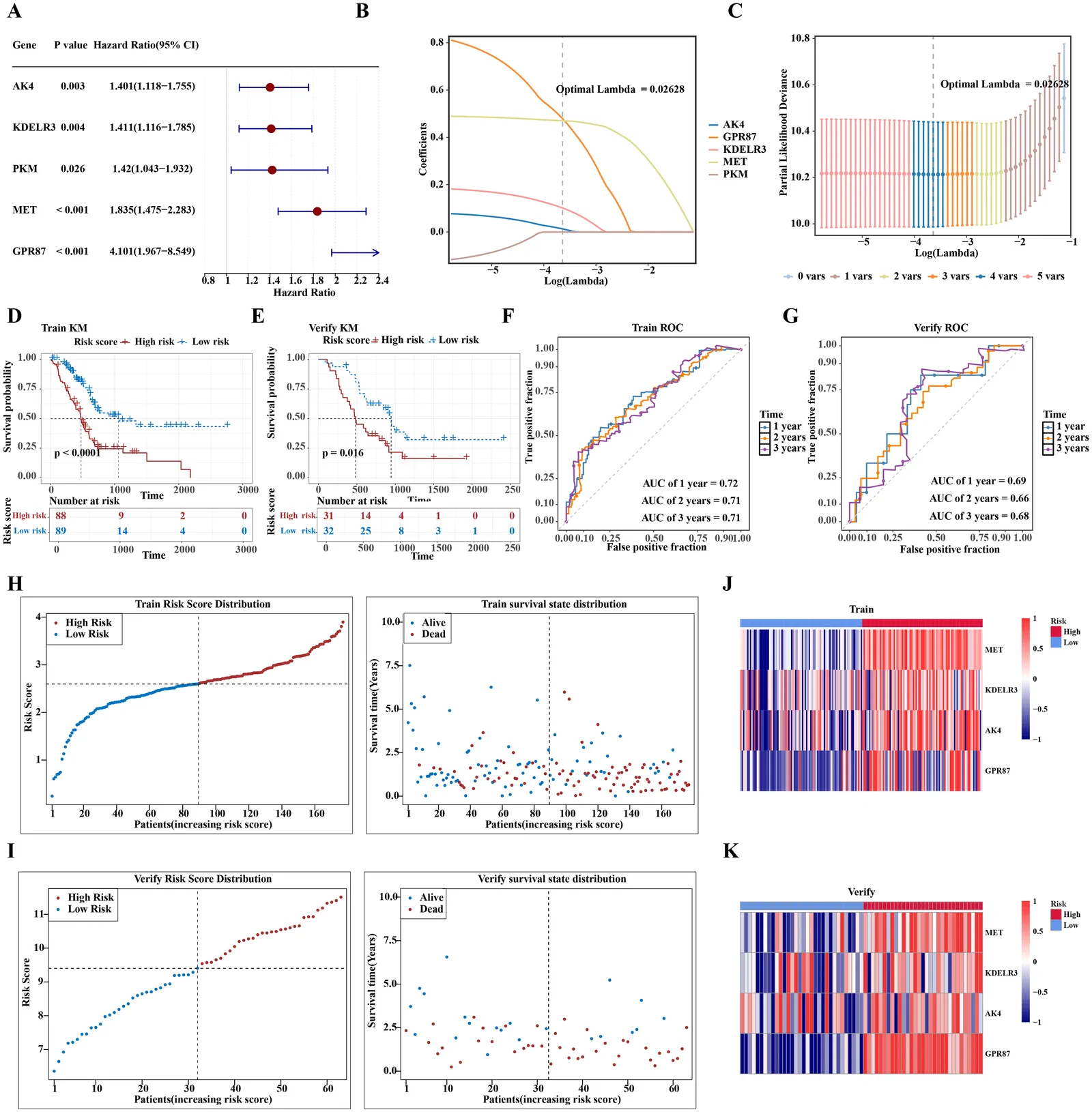
Identification of *MET*, KDEL*R*3, *AK4*, and *GPR87* as key prognostic genes **(A)** Univariate Cox regression forest plot illustrates prognosis-related genes. **(B)** Gene coefficient plot. The x-axis shows the logarithm of lambda values, and the y-axis shows the model error. **(C)** Cross-validation error plot. The x-axis shows the logarithm of lambda values, and the y-axis shows the variable coefficients. As lambda increases, the variable coefficients approach 0. When the optimal lambda is reached, variables with a coefficient of 0 are excluded. **(D)** In TCGA-PAAD, the Kaplan-Meier survival curves indicated that patients in the high-risk group had a significantly lower survival probability than those in the low-risk group. Red represents the high-risk group, blue represents the low-risk group, the horizontal axis represents follow-up time, and the vertical axis represents the survival rate. **(E)** In GSE57495, the Kaplan-Meier survival curves showed that patients in the high-risk group had significantly lower survival probability than those in the low-risk group. Red represents the high-risk group, blue represents the low-risk group, the horizontal axis represents follow-up time, and the vertical axis represents the survival rate. **(F, G)** The constructed risk model exhibited moderate predictive performance in the training and validation sets (area under the curve values exceeding 0.65 at 1, 2, and 3 years). The horizontal axis represents false positives, and the vertical axis represents true positives. **(H)** Risk curves and survival status of patients in the training set. **(I)** Risk curves and survival status of patients in the validation set. **(J)** Expression heatmap of prognostic genes in the high- and low-risk groups of the training set. **(K)** Expression heatmap of prognostic genes in the high- and low-risk groups of the validation set.


Univariate Cox regression analysis (HR ≠ 1 and p < 0.05) and PH assumption (p > 0.05) identified five prognosis-related genes (**Figure 3A**; **Supplementary Table 6**, **7**). These genes were then subjected to LASSO analysis, which identified *MET*, *KDELR3*, *AK4*, and *GPR87* as prognostic genes at lambdamin = 0.02628 (**Figures 3B, C**). In the GSE28735 and GSE62452 datasets, the expression of these four genes was significantly upregulated in PC (p < 0.05), and the overall survival rates of patients in the high-expression groups for each gene were lower than those in the low-expression groups (**Supplementary Figures 1A, B**). Prognostic models were subsequently constructed based on these prognostic genes.

Risk score=MET×0.470+KDELR3×0.101+AK4×0.013+GPR87×0.479

The TCGA-PAAD samples with survival information were categorized into two groups by the median risk score: a high-risk group (88 samples) and a low-risk group (89 samples), using a median risk score of 2.597068 as the cutoff. Similarly,GSE57495 was stratified using its dataset-specific median cutoff (9.40481) into high- (n=31) and low-risk (n=32) groups. In TCGA-PAAD and GSE57495, the KM curves showed that patients in the high-risk group had a lower survival probability (p < 0.05) (**Figures 3D, E**). Furthermore, the results of the ROC analysis for 1-, 2-, and 3-year survival risks revealed that the AUC values of the exploratory risk model were 0.72, 0.71, and 0.71 in TCGA-PAAD dataset, and 0.69, 0.66, and 0.68 in GSE57495 dataset, which suggests that the model has moderate predictive power regarding the survival status of patients with PC (**Figures 3F, G**). As the risk score increased, the risk curves exhibited a gradual increase in mortality among patients with PC (**Figures 3H, I**). The heat map illustrated the expression patterns of prognostic genes, suggesting a potential close correlation between these genes and a poor prognosis in patients with PC (**Figures 3J, K**). Furthermore, applying the TCGA-PAAD risk score cutoff value of 2.597068 to the GSE79668 dataset divided the samples into a high-risk group (33 samples) and a low-risk group (16 samples). The results of this validation set analysis were consistent with those of the training set: the high-risk group had a lower probability of survival, with AUC values of 0.71, 0.73, and 0.81 at 1, 2, and 3 years, respectively (**Supplementary Figures 2A–D**).

### 3.4 Construction of a prognostic nomogram based on independent prognostic factors

**Figure 4:**
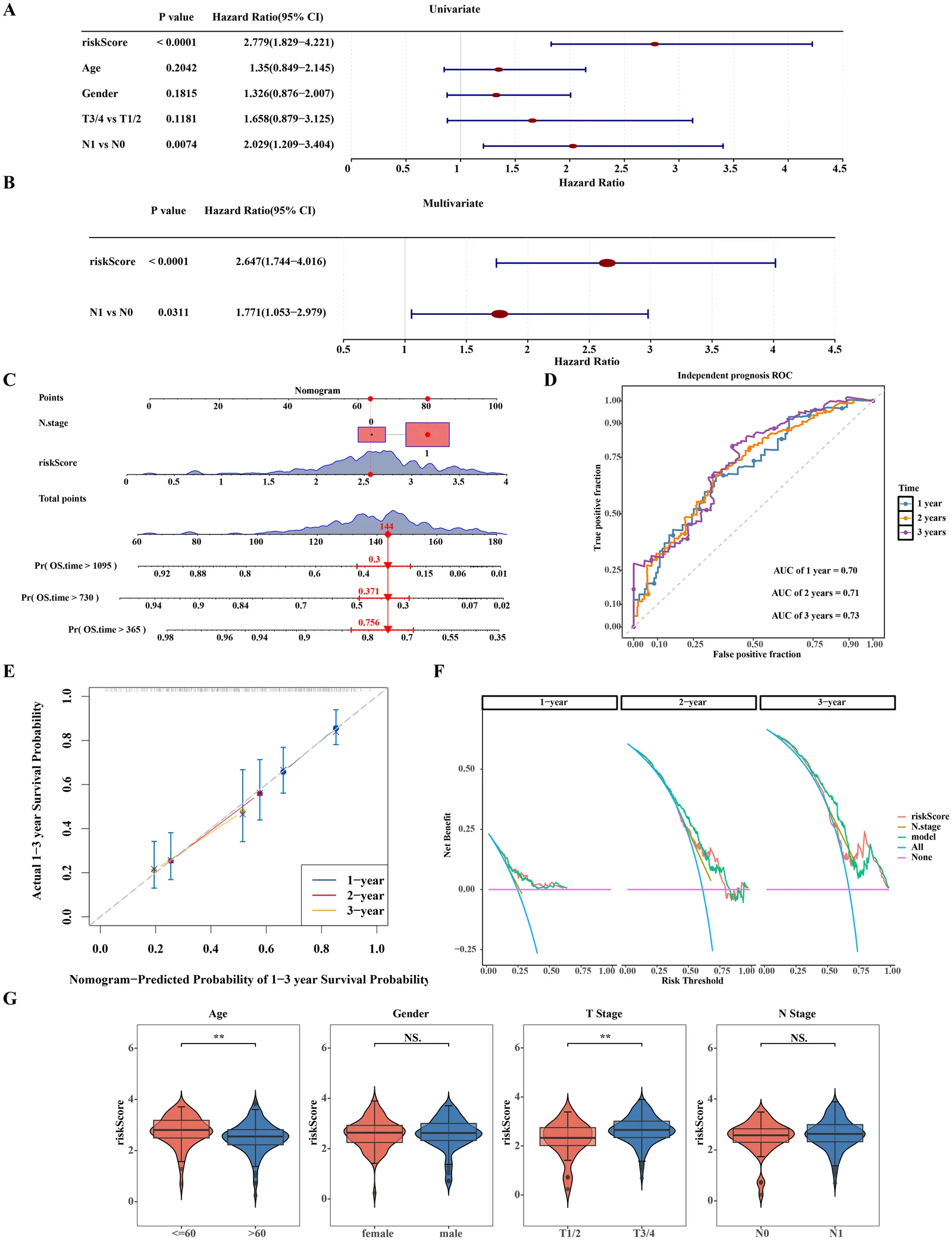
Prognostic nomogram for pancreatic cancer based on independent prognostic factors **(A)** Forest plot of clinical variables from univariate Cox regression analysis. **(B)** Forest plot of clinical variables from multivariate Cox regression analysis. **(C)** Construction of the prognostic nomogram. Each line segment corresponding to a variable is labeled with a scale indicating the variable’s range. In contrast, the length of the segment reflects the magnitude of that factor’s contribution to the outcome. The “Points” shown in the image represent the individual scores for each variable at different values. The “TotalPoints” shown in the image represents the total score obtained by summing the individual scores corresponding to all variable values. **(D, E)** Receiver operating characteristic curve and calibration curve based on the nomogram. **(F)** The 1-, 2-, and 3-year decision curve analysis. The x-axis represents risk thresholds, and the y-axis represents net benefit. **(G)** Violin plot showing differences in the risk scores among subgroups stratified by different clinical features. **p < 0.01 and NS, non-significant.

Independent prognostic analyseswas performed to evaluate whether the risk score could serve as an independent predictor of survival, which is a prerequisite for its potential future utility in clinical prognostic assessment. Univariate Cox regression analysis showed that the risk score and N staging were both significantly associated with patient prognosis (HR > 1, p < 0.05) and met the PH assumption (p > 0.05) (**Figure 4A**; **Supplementary Table 8**). Multivariate Cox regression analysis further demonstrated that, after adjusting for other variables, the risk score and N stage remained significant independent prognostic factors (HR > 1, p < 0.05), and this multivariate model also satisfied the PH assumption (p > 0.05) (**Figure 4B**; **Supplementary Table 9**). A nomogram was developed using the independent prognostic factors (**Figure 4C**). The AUCs for 1-, 2-, and 3-year survival were 0.70, 0.71, and 0.73, respectively. These results indicated that the model exhibited moderate discriminative ability for 1-, 2-, and 3-year survival status, but further validation is required before clinical application. (**Figure 4D**). The calibration curve showed no significant deviation between the model-predicted and observed survival probabilities (**Figure 4E**). According to the DCA, the net benefit of the nomogram exceeded that of a single clinical indicator (N staging) at the majority of the risk thresholds; simultaneously, compared to using risk score alone, the nomogram showed a more robust and higher net benefit at multiple time points (most notably at 2 and 3 years) (**Figure 4F**). A stratified analysis of the risk scores and clinical characteristics indicated that notable differences in risk scores were observed among two clinical characteristics: age (categorized as ≥60 years and <60 years) and T stage (grouped as T1-T2 vs. T3-T4) (p < 0.05) (**Figure 4G**).

### 3.5 GSEA and immune infiltration analysis

**Figure 5:**
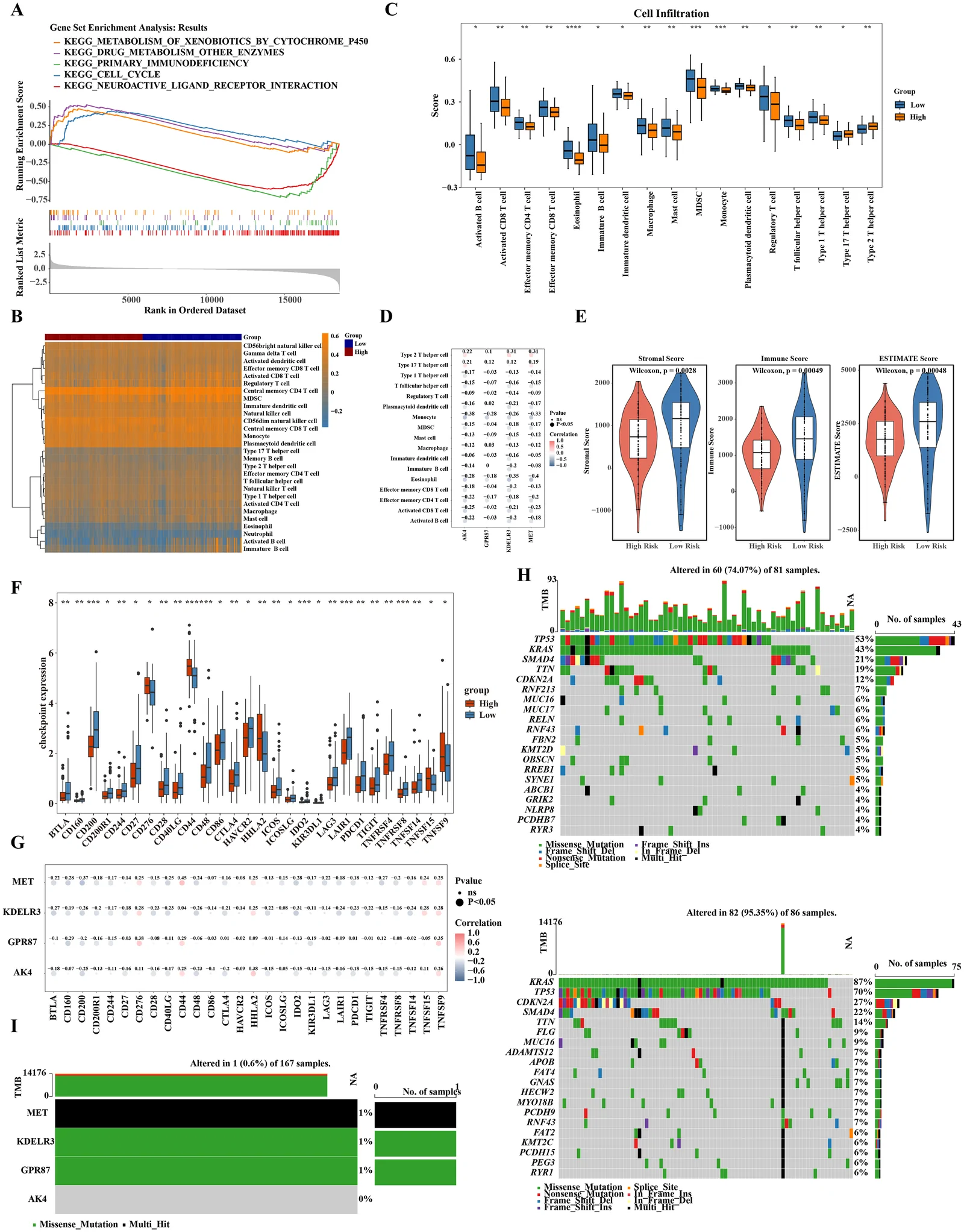
Gene set enrichment analysis (GSEA) and immune infiltration analysis based on the risk model **(A)** GSEA of the high- and low-risk groups in the training set. Part 1: The top five line graphs represent the gene enrichment scores. The vertical axis shows the corresponding running enrichment scores; the line graph shows a peak, representing the enrichment score for this gene set. The genes preceding this peak are the core genes within this gene set. The horizontal axis represents each gene in this gene set, corresponding to the vertical lines in Part 2. Part 2: The barcode-like section represents Hits, with each vertical line corresponding to a gene within the gene set. Part 3: This is a distribution plot of rank values for all genes, with the vertical axis representing the ranked list metric, i.e., the value of the gene’s rank. **(B)** Heatmap of enrichment scores of immune-infiltrating cells in the high- and low-risk groups of the training set. **(C)** Boxplots of differential immune cells between the high- and low-risk groups in the training set. **(D)** Correlation analysis between the prognostic genes and differential immune cells. **(E)** Stromal, immune, and ESTIMATE scores in the high- and low-risk groups. **(F)** Expression profiles of the immune checkpoints in the high- and low-risk groups. **(G)** Correlation between the prognostic genes and differential immune checkpoints. **(H)** Mutation analysis of the high- and low-risk groups. Rows represent genes, and columns represent samples. **(I)** Mutation analysis based on the prognostic genes. Rows represent genes, and columns represent samples. ****p < 0.0001, ***p < 0.001, **p < 0.01, and *p < 0.05.

The GSEA results of the study indicated that the two risk groups exhibited enrichment in 44 functional pathways. Notably, these risk groups revealed significant enrichment for pathways such as KEGG neuroactive ligand-receptor interaction and KEGG cell cycle, as well as KEGG primary immunodeficiency (**Figure 5A**; **Supplementary Table 10**). Furthermore, it displayed the abundance of 28 immune-infiltrating cells (**Figure 5B**). Further analysis identified 17 differential immune cells exhibiting significant variation between the risk groups (p < 0.05). Among them, the infiltration levels of the other 15 differential immune cells were higher in the low-risk group, whereas type 17 T helper cells and type 2 T helper cells were higher in the high-risk group (**Figure 5C**). The correlation results showed that the prognostic genes and differential immune cells exhibited a significant positive correlation between *KDELR3* and type 2 T helper cells (cor = 0.31, p < 0.05). The highest negative correlation between *MET* and eosinophil (cor = -0.40 and p < 0.05) (**Figure 5D**). In TCGA-PAAD of PC samples, the high-risk group had lower stromal (p < 0.0028), immune (p < 0.00049), and ESTIMATE (p < 0.00048) scores than the low-risk group (**Figure 5E**). Moreover, the 28 immune checkpoints were significantly different between the two risk groups (p < 0.05) (**Figure 5F**). CD44 and *MET* demonstrated the highest significant positive correlation (|cor| > 0.45 and p < 0.05); however, the strongest significant negative correlation was noted between CD200 and *MET* (cor = -0.37, p < 0.05) (**Figure 5G**). In the high-risk group, *KRAS*, *TP53*, and *CDKN2A* exhibited the highest mutation frequency. In the low-risk group, *TP53*, *KRAS*, and *SMAD4* exhibited the highest mutation rates (**Figure 5H**). *KDELR3*, *AK4*, and GPR*8*7 were primarily mutated via missense mutations (**Figure 5I**).

### 3.6 lncRNA-miRNA-mRNA regulatory network

The lncRNA-miRNA-mRNA network was constructed, and there were 26 miRNAs and 50 lncRNAs with regulatory relationships to *MET* and *AK4* mRNA. *MET* regulates RMRP via hsa-miR-1-3p, and *AK4* regulates AC005261.1 via hsa-miR-15a-5p (**Supplementary Figure 3A**; **Supplementary Table 11**).

### 3.7 Drug sensitivity analysis

**Figure 6:**
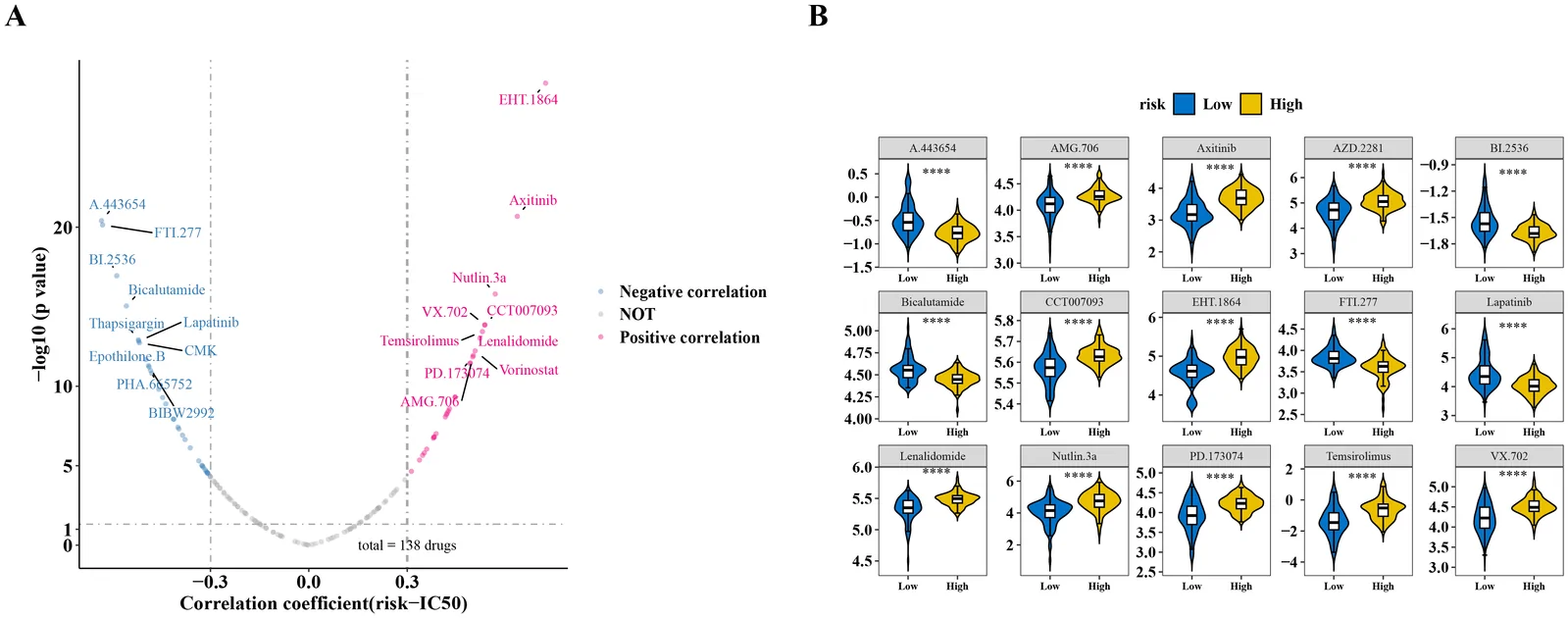
Drug sensitivity analysis **(A)** Drug screening based on the correlation between drug IC_50_ values and risk scores. **(B)** Comparison of differences in the IC_50_ values of common chemotherapeutic drugs between the high- and low-risk groups using the Wilcoxon test (p < 0.05), with the top 15 drugs displayed. ****p < 0.0001.

The correlation between the IC_50_ of 138 common chemotherapy drugs and the risk score was analyzed. Computational prediction identified 60 drugs with a strong correlation with the risk score; the risk score was computationally predicted to be positively associated with sensitivity to 28 drugs, such as EHT.1864 and Axitinib. The risk score was negatively correlated with sensitivity to 32 drugs, such as A.443654 and FTI.277 (**Figure 6A**). The IC_50_ values were used to assess the sensitivity of PC to common drugs. Significant differences between the top 15 (sorted by p-value) drugs within the groups are shown in **Figure 6B**. Among these, drug sensitivity analysis predicted lower IC50 values for A.443654, BI.2536, bicalutamide, FTI.277, and lapatinib in the high-risk group, whereas AMG.706, AZD.2281, axitinib, CCT007093, EHT.1864, lenalidomide, Nutlin.3a, PD.173074, temsirolimus, and VX.702 showed lower IC50 values in the low-risk group (p<0.05).It was noted that the above results were based on computational predictions and required further experimental verification.

### 3.8 Preliminary functional characterization of *GPR87*, a high-weight component of the prognostic model, in pancreatic cancer cells

**Figure 7:**
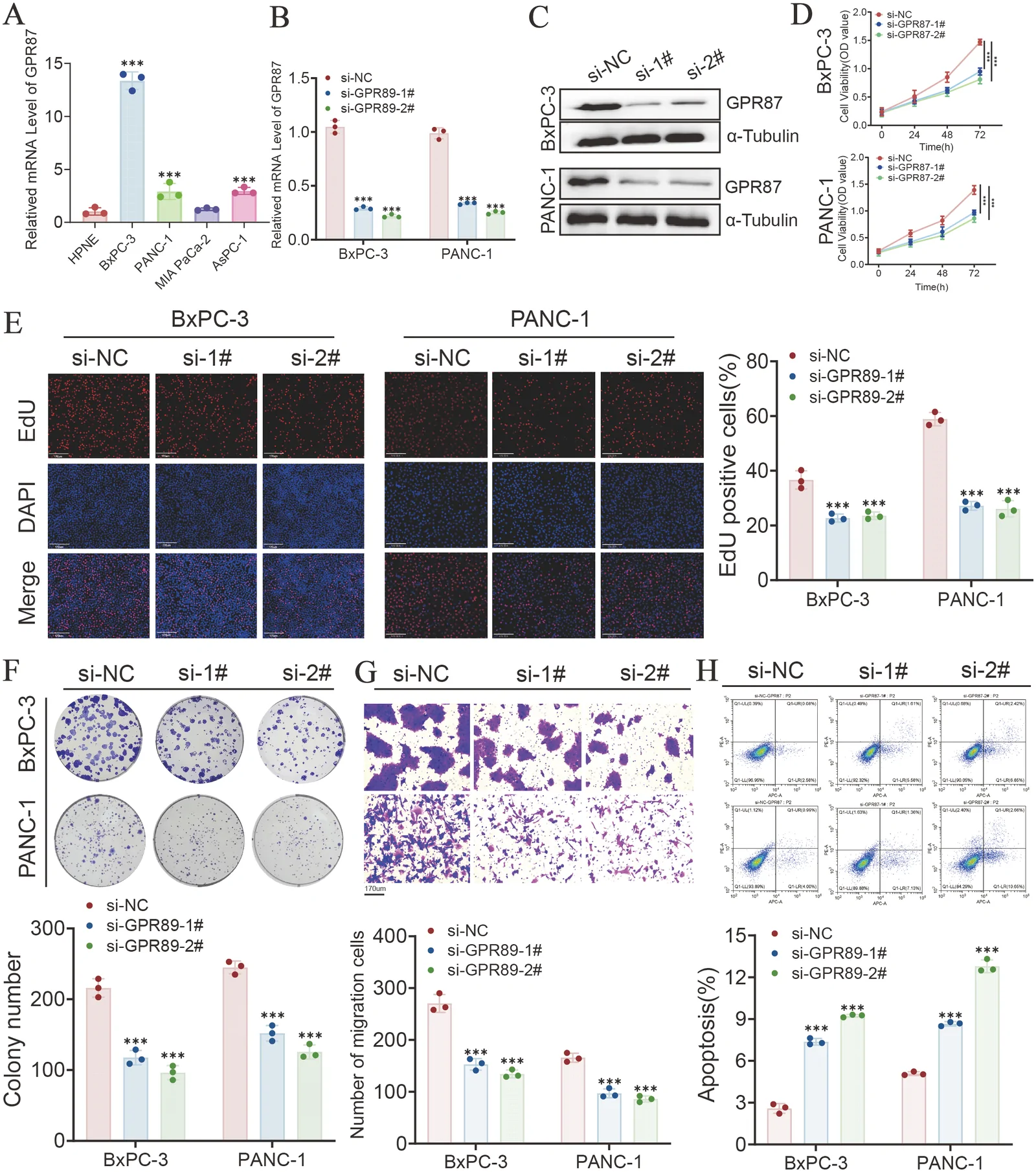
*In vitro* validation of *GPR87* knockdown-mediated inhibition of malignant biological behaviors in pancreatic cancer (PC) cells **(A)** RT-qPCR analysis of *GPR87* mRNA expression in four PC cell lines and one normal pancreatic cell line (*n* = 3 biologically independent samples). **(B)** RT-qPCR verification of *GPR87* knockdown efficiency by small interfering RNAs (siRNAs) in BxPC-3 and PANC-1 PC cell lines (*n* = 3 biologically independent samples). **(C)** Examine the GPR87 knockdown efficiency in two cell lines by Western blot (*n* = 3 biologically independent samples). **(D)** CCK-8 proliferation assay for evaluating the effect of *GPR87* knockdown on the proliferative capacity of PC cells (*n* = 3 biologically independent samples). **(E)** EdU incorporation assay for assessing the effect of *GPR87* knockdown on PC cell proliferation (*n* = 3 biologically independent samples). **(F)** Colony formation assay for determining the effect of *GPR87* knockdown on the colony-forming ability of PC cells (*n* = 3 biologically independent samples). **(G)** Transwell migration assay for investigating the influence of *GPR87* knockdown on the migratory capacity of PC cells (*n* = 3 biologically independent samples). **(H)** Flow cytometry apoptosis assay for detecting the effect of *GPR87* knockdown on the apoptotic potential of PC cells (*n* = 3 biologically independent samples). ns > 0.05, *< 0.05, **p < 0.01 and ***p < 0.001.

Utilizing the previously established risk scoring formula (Risk score = MET × 0.470 + KDELR 3 × 0.101 + AK4 × 0.013 + GPR87 × 0.479), we further investigated the role of *GPR87* in the onset and progression of PC. *GPR87* was selected as the primary gene for experimental validation due to its highest risk coefficient, relatively unexplored function in PC compared with MET, and potential involvement in glycolytic-immune regulation. Research indicates that *GPR87* is significantly overexpressed in the PC cell lines BxPC-3, PANC-1, and AsPC-1 compared to normal pancreatic cells. In contrast, the expression of *GPR87* in MIA-PACA2 PC cells does not differ significantly from that observed in normal pancreatic cells (**Figure 7A**).

To elucidate the functional role of *GPR87* in PC progression, we used siRNAs to downregulate *GPR87* in BxPC-3 and PANC-1 cells. RT-qPCR and WB results confirmed the successful knockdown of *GPR87* in BxPC-3 cells (**Figures 7B, C**). Subsequently, results from the CCK8 proliferation assay and EdU incorporation assay demonstrated that *GPR87* knockdown significantly inhibited the proliferative capacity of BxPC-3 and PANC-1 cells (**Figures 7D, E**). In addition, the colony formation and Transwell migration assays further established that *GPR87* silencing markedly reduced the colony-forming and migratory abilities of these cell lines (**Figures 7F, G**). Furthermore, flow cytometry analysis of apoptosis indicated that GPR87 knockdown facilitated the apoptotic process in BxPC-3 and PANC-1 cells (**Figure 7H**). In addition, *MET* also bears a high weight value in the risk scoring formula. We performed IHC staining to detect *MET* expression in paired tumor and normal pancreatic cancer tissues. The results showed that *MET* was highly expressed in pancreatic cancer tissues (**Supplementary Figure 3B**).

**Figure 8:**
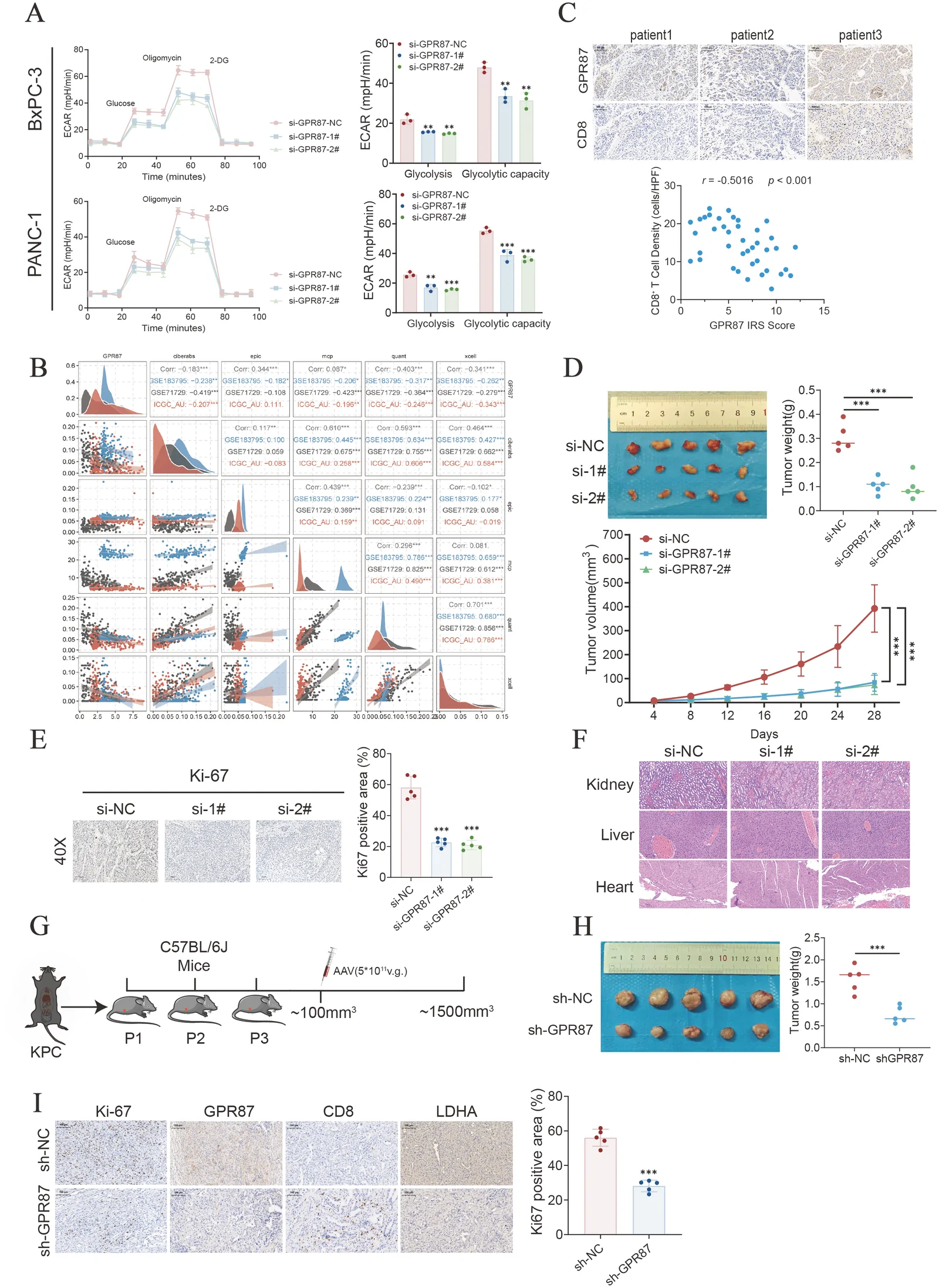
The potential role of *GPR87* in glucose metabolism and immune infiltration, *in vivo* validation of *GPR87* knockdown-mediated suppression of pancreatic cancer tumor growth **(A)** To detect ECAR after *GPR87*- knockdown (*n* = 3 biologically independent samples). **(B)** Correlation between *GPR87* expression and CD8^+^ T cell infiltration in three GEO transcriptomic cohorts analyzed by multiple immune deconvolution algorithms. **(C)** Immunohistochemical validation of *GPR87* and CD8 expression in tumor tissues from the same pancreatic cancer patients. **(D)** Subcutaneous xenograft assay in nude mice using small interfering RNA (siRNA)-transfected *GPR87*-knockdown and negative-control BxPC-3 cells. Tumor weights in the *GPR87*-knockdown and control groups. Dynamic changes in the tumor volumes of nude mice after cell inoculation (*n* = 4 per group). **(E)** Ki-67 expression in tumor sections from the *GPR87*-knockdown and control groups (*n* = 4 per group). **(F)** Hematoxylin and eosin staining of the heart, liver, and kidney tissues from nude mice in the *GPR87*-knockdown and control groups (20×). **(G)** KPC-derived syngeneic subcutaneous xenograft model. **(H)** Schematic diagram of tumor volume in mice after GPR87 knockdown via AAV injection. **(I)** Immunohistochemistry was performed to detect the expression of *Ki-67*, *GPR87*, *LDHA* and *CD8* in tumor tissues from the two groups. ns > 0.05, *< 0.05, **p < 0.01 and ***p < 0.001.

To preliminarily explore *GPR87*’s potential role in pancreatic cancer glucose metabolism and immunity, we found that *GPR87* knockdown decreased ECAR and impaired glucose uptake in two pancreatic cancer cell lines, accompanied by reduced expression of HK2, GLUT1 and LDHA. (**Figure 8A**; **Supplementary Figures 3C, D, E**). We analyzed three transcriptomic cohorts (ICGC-AU, GSE183795, GSE717295) with five immune-infiltration algorithms to assess the relationship between *GPR87* expression and CD8^+^ T cell infiltration (**Figure 8B**). The analyses revealed a significant negative correlation between *GPR87* expression and CD8^+^ T cell abundance. Immunohistochemistry of tumor specimens from pancreatic cancer patients corroborated this observation (*r* = -0.5016, *p* < 0.001) (**Figure 8C**). These findings are correlative in nature and do not establish that *GPR87* directly regulates CD8+ T cell infiltration.

Building on these *in vitro* findings, we performed subcutaneous tumor formation experiments by inoculating nude mice with *GPR87* knockdown BxPC-3 cells and negative-control BxPC-3 cells. The results indicated that the volume of pancreatic tumors in the *GPR87* knockdown group was significantly smaller than in the control group, and tumor weight was also markedly reduced (**Figure 8D**). This confirms that *GPR87* knockdown effectively inhibits PC growth *in vivo*. Immunohistochemical staining for Ki-67 on tissue sections from three groups of transplanted tumors revealed a significant reduction in the number of Ki-67-positive cells in the *GPR87* knockdown group (**Figure 8E**), thereby further substantiating its inhibitory effect on PC proliferation. In addition, there were no significant differences in the hematoxylin and eosin staining results of the heart, liver, and kidney tissues among the three groups of nude mice (**Figure 8F**), which suggests that this intervention exhibits good safety in this model. To further investigate whether *GPR87* knockdown affects CD8^+^ T cell infiltration in an immunocompetent tumor microenvironment, we established a syngeneic subcutaneous tumor model in C57BL/6 mice using pancreatic tumor tissues harvested from KPC mice (**Figures 8G, H**). Immunohistochemical staining demonstrated that *GPR87* knockdown tumors exhibited significantly increased CD8^+^ T cell infiltration compared with the control group. Furthermore, we found that upon *GPR87* suppression, both Ki-67 positivity and *LDHA* expression were downregulated in tumor tissues (**Figure 8I**). These findings provide preliminary *in vivo* evidence supporting a potential link between *GPR87* expression and CD8^+^ T cell abundance in the tumor microenvironment.

Furthermore, the results indicate that *GPR87* is highly expressed in PC. The knockdown of *GPR87* inhibits the proliferation and migration of PC cells *in vitro* while promoting their apoptosis. In addition, *in vivo* assays further verify that silencing *GPR87* markedly impedes PC progression, accompanied by downregulated *LDHA* expression and elevated CD8 levels. This study offers preliminary experimental evidence for the potential involvement of *GPR87* in PC progression, as well as its regulatory roles in tumor metabolism and antitumor immunity, which merits further in-depth exploration.

## 4 Discussion

PC is a highly aggressive cancer with a dismal prognosis, necessitating the development of new therapeutics and prognostic indicators (41). Its immunosuppressive microenvironment is characterized by a dense extracellular matrix, hypoxia, impaired angiogenesis, and altered immune cell infiltration, which drives disease progression and therapy resistance (42, 43). In this TME, T cell infiltration is reported to be often reduced, and T cell function may be impaired, with metabolic alterations, including enhanced glycolysis, potentially contributing to an immunosuppressive environment (42, 43). In this study, we combined T-RGs and G-RGs, investigated potential cross-regulatory interactions between immunity and metabolism, identified important molecules linked to PC prognosis, and developed a prognostic model.

This study used T cell immune infiltration analysis and WGCNA to integrate DEGs from multiple independent datasets with T-RGs and G-RGs. Ultimately, five core candidate genes were identified, and KEGG and GO functional enrichment analyses were conducted. The findings suggested that these candidate genes are associated with various fundamental metabolic functions within the organism. In addition, through survival analysis and LASSO regression, four independent prognostic risk genes were identified: *MET*, *KDELR3*, *AK4*, and *GPR87*.

The *AK4* gene encodes a mitochondrial enzyme that regulates the balance of adenine and guanine nucleotides (44). It is linked to oncogenesis in various cancers (45, 46); however, research on *AK4* in PC is scarce. One study showed elevated *AK4* levels in pancreatic tumors associated with a worse prognosis (47). Conversely, another study found no prognostic value of *AK4* expression in PC survival (48). Therefore, further large cohort studies are required to define the expression of *AK4* in PC. In terms of functional mechanisms, previous studies have shown that *AK4* may promote the progression of ovarian cancer through nucleotide and glucose metabolic pathways (49). As a stress-responsive protein, *AK4* inhibits apoptosis by interacting with mitochondrial ADP/ATP translocase (50). In our study, glycolysis-related pathways were significantly enriched in the high-risk group, as indicated by the GSEA results. We speculate that *AK4* may influence immune cell function in the TME through energy metabolism. Whether *AK4* affects T cell metabolic adaptability and functional states remains to be determined. Further studies are needed to clarify its potential mechanisms in PC.

*KDELR3* is a member of the KDEL (Lys-Asp-Glu-Leu) receptor family (51). Previous studies have indicated that *KDELR3* functions as an adaptive gene in response to the accumulation of unfolded or misfolded proteins within the endoplasmic reticulum (51). Shanshan Liu et al. demonstrated, using a novel endoplasmic reticulum stress (ERS)-related risk signature, that high ERS risk enhances glycolytic activity and nucleotide metabolism while suppressing antitumor immunity (52). The results reported here are consistent with that conclusion. Notably, *KDELR1*, a member of the same family, regulates T cell homeostasis by modulating protein phosphatase 1 (53). Given the structural and functional similarities between *KDELR3* and *KDELR1*, *KDELR3* may share related biological functions. Future studies should clarify the specific mechanism by which *KDELR3* mediates metabolic reprogramming and T cell immune regulation in PC.

*MET*, also known as c-MET or HGFR, is a proto-oncogene that encodes a receptor tyrosine kinase expressed in epithelial and endothelial cells (54). It plays a critical role in oncogenesis by promoting tumor progression via immunosuppression. Studies have shown that HGF-stimulated *MET* signaling significantly enhances glycolysis in tumor cells (53). This suggests that *MET* could potentially participate in TME remodeling through glycolysis-associated metabolic alterations. Han et al. further reported that *MET* activation creates an immunosuppressive microenvironment in PC. It facilitates GITR^+^ Treg-driven immune escape via the MET–IL-23–STAT4 axis (55). These findings suggest that *MET* may be associated with the interplay between glycolytic alterations and T cell dysfunction. Therefore, *MET* may represent a potential candidate gene contributing to prognostic modeling in PC. IHC analysis of clinical pancreatic cancer specimens revealed high *MET* protein expression, which provides tissue-level evidence for its upregulation at the transcriptomic level. Further functional experiments are required to clarify MET-mediated crosstalk between glycolysis and immunity.

*GPR87* encodes a G protein–coupled receptor (GPCR), which is a large family of membrane proteins that detect extracellular signals and modulate intracellular processes via G protein–mediated pathways (56). Notably, GPCR signaling and its regulatory molecules play vital roles in the activation, homeostasis, and functional maintenance of T cells (57). Multiple studies have implicated *GPR87* in diverse physiological and pathological contexts. In terms of metabolic regulation, these studies have confirmed that *GPR87* promotes glycolysis and induces mitochondrial damage. This exacerbates renal interstitial fibrosis and may accelerate the progression of chronic kidney disease (58). In PC, Li et al. found that the overexpression of *GPR87* significantly enhanced tumor cell proliferation, angiogenesis, and resistance to gemcitabine-induced apoptosis. In contrast, *GPR87* silencing suppressed these effects (59). Our findings are consistent with these results. We confirmed that *GPR87* is highly expressed in PC cells. *GPR87* knockdown inhibits the malignant behaviors of cancer cells and restricts the progression of subcutaneous xenografts. However, unlike previous studies that only reported the pro-tumor function of *GPR87*, our study included *GPR87* alongside *MET*, *KDELR3*, and *AK4* in a prognostic risk model. This model was constructed based on T-RGs and G-RGs. Risk stratification based on this model showed that the high-risk group (characterized by a higher composite risk score reflecting the combined expression levels of *MET*, *KDELR3*, *AK4*, and *GPR87*) had fewer CD8^+^ T cells, along with higher proportions of regulatory T cells and Th2 cells. Subsequent cellular experiments demonstrated that *GPR87* knockdown reduced extracellular acidification rate, glucose uptake, and the expression of several key glycolytic genes, providing more robust evidence that GPR87 modulates glycolytic reprogramming in pancreatic cancer cells. Consistently, *GPR87* expression was negatively correlated with CD8^+^ T cell infiltration. Based on these correlative findings, we speculate that GPR87 may be associated with the tumor microenvironment, potentially through glycolysis-related metabolic alterations and T cell-associated processes. It should be emphasized that these observations are only derived from transcriptome-based immune infiltration algorithms and IHC correlation analyses, and cannot confirm that *GPR87* directly regulates CD8^+^ T cells or immune suppression. However, the association between *GPR87* and CD8^+^ T cell infiltration or immune features is supported by transcriptomic deconvolution, IHC, and preliminary syngeneic models, but does not establish direct immune regulation. Further functional studies are required to clarify its role in T cell recruitment or activity (60).

Moreover, we did not comprehensively profile glycolysis-related signaling pathways in this study. The functional crosstalk among *GPR87*, glycolysis and T cell immunity remains merely a speculative hypothesis. Future studies using T cell co-culture systems, conditional knockout models and glycolysis pathway detection are required to clarify whether and how *GPR87* modulates glycolytic reprogramming and T cell infiltration.

The risk scoring model developed from the four aforementioned genes effectively differentiated between the high- and low-risk patient subgroups in the training and validation sets. The overall survival duration in the high-risk group is significantly reduced, while the model’s AUCs at 1 and 3 years exceed 0.6, indicating moderate predictive capability in this exploratory analysis. Clinical correlation analysis demonstrated a significant association between the risk score and N stage, implying that this model may reflect the tumor’s metastatic potential. Further mechanistic exploration via GSEA indicated that the high-risk group was significantly enriched in pathways such as “neuroactive ligand-receptor interaction” and “cell cycle.” The former pertains to intricate intercellular communication and may relate to the neural infiltration in PC and TME remodeling. The latter suggests that the high-risk group exhibits a direct response to the malignant biological characteristics of tumor cells. Immune infiltration analysis revealed notable differences in the abundance of various immune cell types between the high- and low-risk groups. Specifically, the proportions of CD8^+^ T cells and effector memory CD4^+^ T cells were reduced in the high-risk group, suggesting a potentially less active antitumor immune landscape that may be associated with the poorer prognosis observed in this cohort. This finding suggests that prognostic genes could influence the outcomes in patients with PC by modulating the TME immune landscape. Furthermore, drug sensitivity analysis identified 60 chemotherapy or targeted agents that were significantly correlated with the risk score. Notably, the high-risk group exhibited lower predicted IC50 values for 28 drugs (including several DNA synthesis inhibitors), while showing higher predicted IC50 values for another 32 drugs (such as certain kinase inhibitors). Although experimental validation is still needed, these insights provide hypothesis-generatingcomputational biological evidence to support the development of personalized treatment strategies for patients with PC across different risk subgroups.

The four-gene prognostic model constructed in this study represents an exploratory analytical framework with potential hypothesis-generating value, rather than a clinically validated tool ready for direct application in PC management. This risk score may serve as a supplementary tool to TNM staging and help identify high-risk patients who may require more aggressive therapeutic interventions. Meanwhile, risk groups differed markedly in immune infiltration and immune checkpoint levels. These exploratory findings raise the hypothesis that risk stratification may reflect differential immunotherapy responsiveness, which requires prospective clinical validation before informing treatment selection. In future prospective validation studies, RT-qPCR-based measurement of *MET*, *KDELR3*, *AK4*, and *GPR87* expression in clinical specimens may offer a cost-accessible approach for risk score calculation. However, standardization of detection protocols and prospective multicenter validation are required to confirm the model’s predictive performance and establish standardized risk score thresholds across diverse patient populations before any clinical implementation. In addition, the feasibility of non-invasive dynamic risk monitoring using liquid biopsy approaches warrants further investigation. Furthermore, Luo et al. (61) recently demonstrated that wider clinical application of precision oncology requires integrated real-world data, standardized diagnostic platforms, and optimized solutions for the limitations of current diagnostic techniques. Accordingly, future research should focus on prospective validation, calibration in independent cohorts, and cost-effectiveness analyses, with the ultimate goal of incorporating this biomarker into routine clinical practice.

This study primarily relies on a retrospective analysis of public databases and select phenotypic experiments, which presents certain limitations. First, this retrospective study relied on public transcriptomic databases. Although validated in two GEO cohorts (GSE57495 and GSE79668), independent analyses avoided data integration-related bias, but the lack of prospective clinical cohorts from diverse platforms limits generalizability. The model showed moderate discrimination (AUC 0.68–0.73) and requires further validation before clinical application. Second, this study focused on T-RGs and G-RGs. Nevertheless, only preliminary experiments were performed to validate *GPR87*, while no expression or functional assays were conducted for *MET*, *KDELR3* and *AK4*. The prognostic performance of these three genes is unstable across different datasets, and their underlying mechanisms mediating metabolism-immune crosstalk remain unclear. In addition, we only evaluated CD8^+^ T cell infiltration via bioinformatic analysis and IHC staining. Markers related to T cell activation and exhaustion, including Granzyme B, IFN-γ and PD-1, were not detected. Flow cytometry and T cell co-culture experiments were not performed either, so the effector function of CD8^+^ T cells cannot be confirmed directly. Moreover, T-cell-deficient nude mice were applied for *in vivo* experiments, which fail to support the exploration of T cell-dependent immune mechanisms. The syngeneic C57BL/6 model provided preliminary evidence linking *GPR87* suppression with increased CD8^+^ T cell infiltration and reduced glycolytic genes, but cannot prove direct T cell regulation. Flow cytometry, depletion, and adoptive transfer studies are needed for mechanistic validation. Besides, key glycolysis signaling pathways were not explored in this study, and the mechanistic link remains unclarified. Drug sensitivity analysis was merely derived from computational prediction without experimental validation. Furthermore, multiple testing correction was not conducted in immune infiltration, immune checkpoint and drug sensitivity analyses, which may cause false-positive results. Finally, patients were not stratified according to KRAS mutation status, and whether KRAS alteration affects model performance and *GPR87* function remains unknown.

Future perspectives are listed as follows. The critical next step is validation in independent multicenter clinical cohorts with diverse KRAS mutation subtypes, to validate and calibrate this exploratory prognostic model Larger clinical samples and complete survival data will be used to confirm the prognostic value of *MET*, *KDELR3* and *AK4*, and relevant functional experiments will be implemented. Gene knockdown or overexpression assays combined with metabolic detection and T cell co-culture models will be adopted to clarify the specific roles of these genes in pancreatic cancer metabolism-immune regulation network. The immunodeficient model limitation was partially addressed by validation in KPC syngeneic models; comprehensive T-cell functional analyses remain for future studies. Representative predicted drugs will be selected for *in vitro* drug sensitivity assays to verify actual drug responses among different risk subgroups. The current use of dataset-specific median cutoffs, while methodologically justified for exploratory cross-cohort analysis, highlights the need for cutoff standardization as a prerequisite for clinical translation. Moreover, key molecules and drug-sensitive signatures identified in this study will be validated in external independent cohorts to improve the reliability and reproducibility of our findings.

## 5 Conclusion

In summary, this study employed bioinformatics methods to integrate two critical perspectives: T cells and glycolysis. It identified four genes with independent prognostic importance in PC (*MET*, *KDELR3*, *AK4*, and *GPR87*) and developed an exploratory risk scoring model that showed moderate predictive performance. Subsequent investigations revealed differences in potential pathways and T cell infiltration patterns between high- and low-risk populations as classified by this model. In addition, the study preliminarily explored the model’s potential utility in predicting drug responses, which requires further experimental and clinical validation. Finally, *in vitro* and *in vivo* experiments demonstrated that *GPR87* plays a significant role in promoting the malignant behavior of PC cells. *GPR87* may also be involved in glucose metabolism and immune-related processes in pancreatic cancer.

## Data Availability

The original contributions presented in the study are included in the article/**Supplementary Material**. Further inquiries can be directed to the corresponding author.
