# Supplementary material for: Glycolysis and T cell-associated gene signature predicts prognosis and therapeutic responses in pancreatic cancer
Source: Front Immunol. 2026 Aug 28;17:1918556. doi: 10.3389/fimmu.2026.1918556 (PMC13562267; doi:10.3389/fimmu.2026.1918556)
Supplement: Supplementary file 1 [file DataSheet1.pdf]

PANC-1

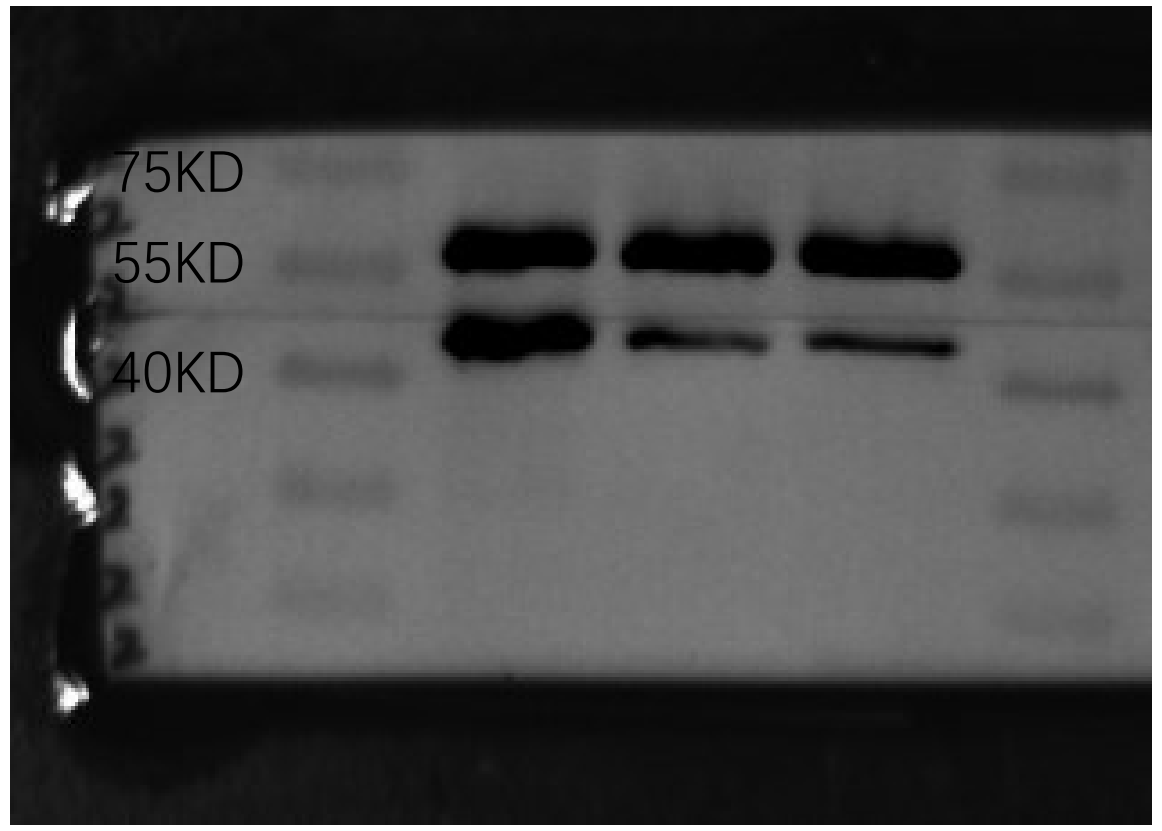

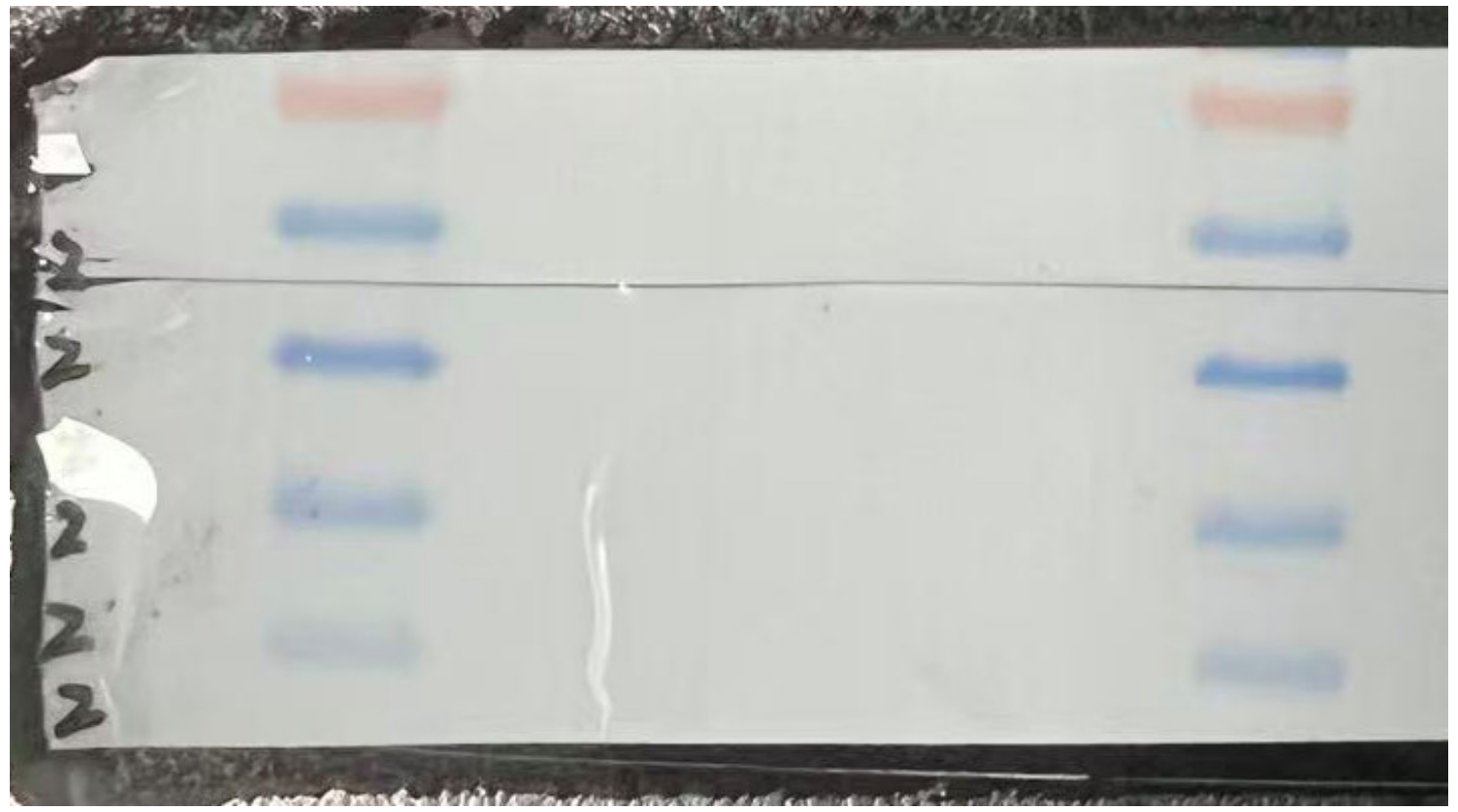

α-Tubulin（55KD）
GPR87（40KD）

BXPC-3

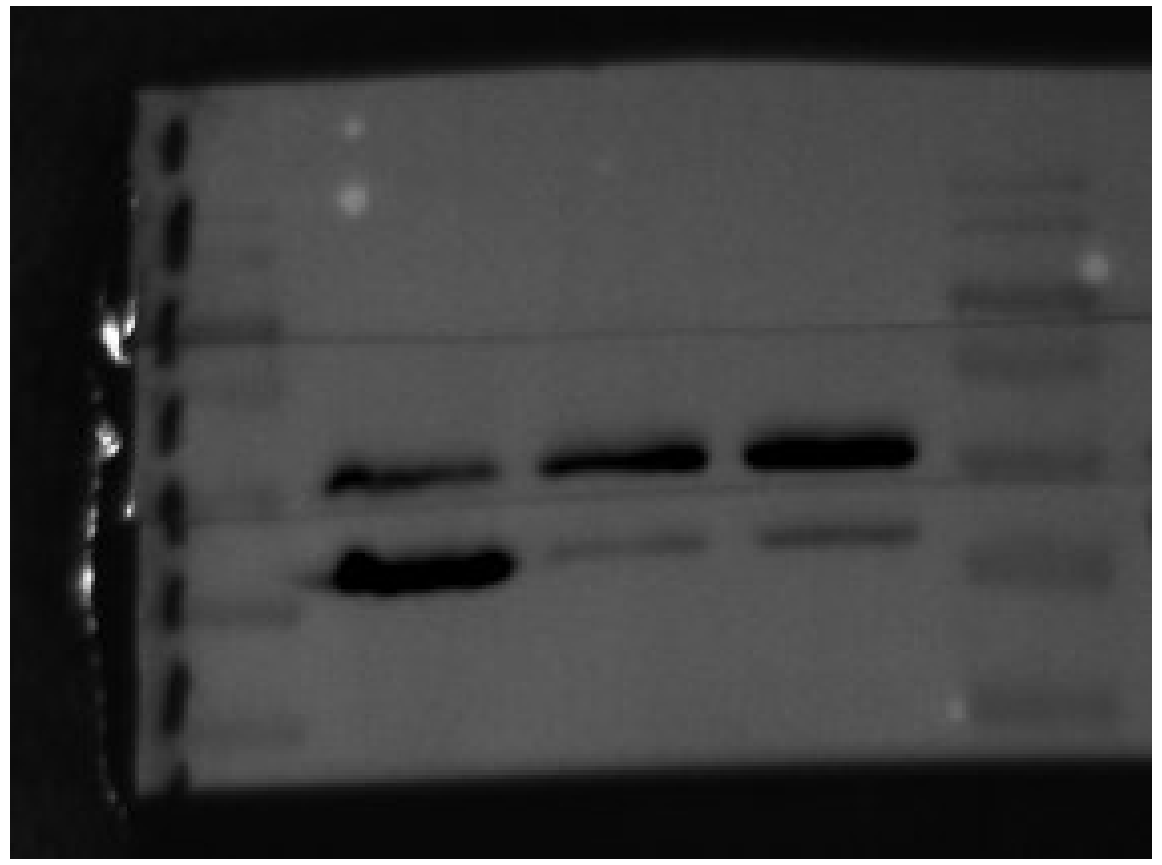

α-Tubulin（55KD）
GPR87（40KD）

BXPC-3

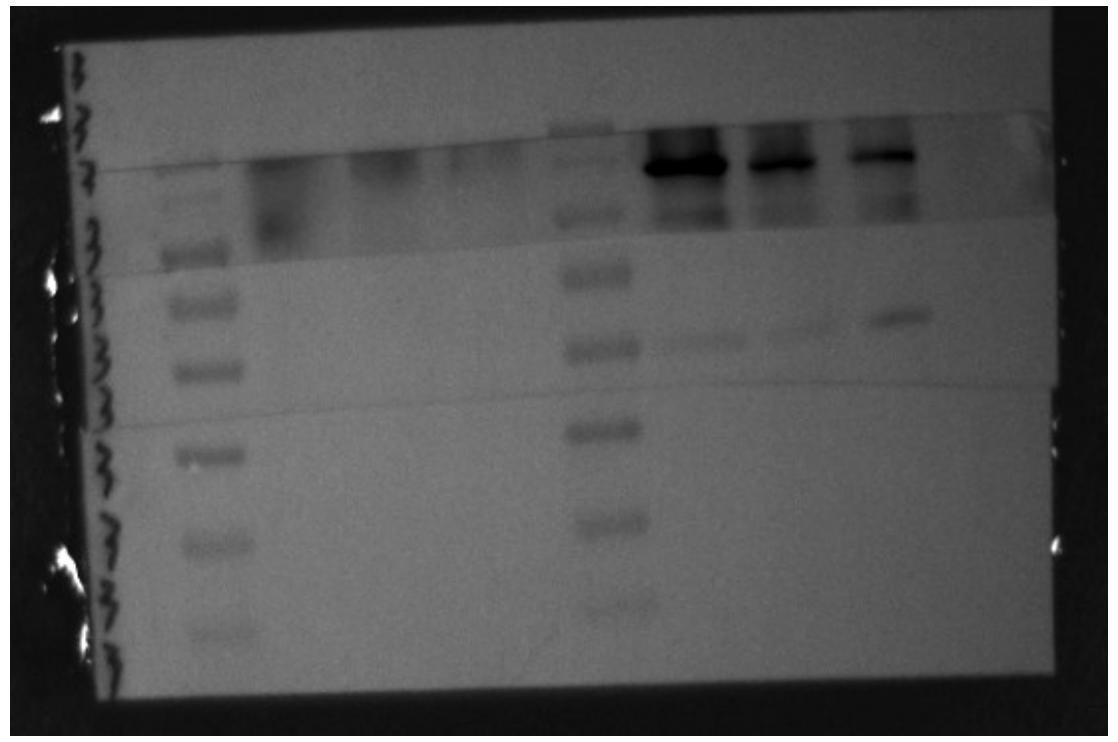

HK2
α-Tubulin（55KD）
