# Supplementary material for: Glycolysis and T cell-associated gene signature predicts prognosis and therapeutic responses in pancreatic cancer
Source: Front Immunol. 2026 Aug 28;17:1918556. doi: 10.3389/fimmu.2026.1918556 (PMC13562267; doi:10.3389/fimmu.2026.1918556)
Supplement: Supplementary file 2 [file Table1.docx]

Supplementary Material

# 1 Supplementary Figures and Tables

## 1.1 Supplementary Figures

**Supplementary Figure 1.** Expression of four prognostic genes in pancreatic cancer **(A)** and survival analysis **(B)** in the GSE28735 and GSE62452 datasets.

**Supplementary Figure 2.** Results of validating the prognostic model using a TCGA-PAAD risk score threshold of 2.597068 in the GSE79668 dataset, including Kaplan-Meier survival curves **(A)**, time-dependent ROC curves **(B)**, distribution of risk scores and survival status **(C)**, and heatmaps of prognostic gene expression in high- and low-risk groups **(D)**.

**Supplementary Figure 3.** **(A)** lncRNA-miRNA-mRNA regulatory networks associated with MET and AK4. **(B)** Immunohistochemistry of MET in pancreatic cancer tumor tissues and normal tissues. **(C)**Glucose uptake assay (*n* = 3 biologically independent samples). **(D)** The expression levels of key glycolytic genes were detected by RT-qPCR (*n* = 3 biologically independent samples). **(E)** Western‑blot images of HK following GPR87 knockdown in BxPC‑3 cells.

## 1.2 Supplementary Tables

Supplementary tables for this project have been submitted as Excel files in the supplementary materials; below are the titles of these supplementary tables.

**Supplementary Table 1** Primer and small interfering RNA (siRNA) sequences employed for qRT-PCR and gene knockdown.

**Supplementary Table 2** Results of correlation analysis among different immune cells.

**Supplementary Table 3** List of 1,752 T-cell-related genes (T-RGs)

**Supplementary Table 4** GO enrichment analysis of candidate genes.

**Supplementary Table 5** KEGG enrichment analysis of candidate genes.

**Supplementary Table 6** Results of univariate proportional hazard (PH) hypothesis tests.

**Supplementary Table 7** Results of the multivariate proportional hazards (PH) hypothesis test.

**Supplementary Table 8** Univariate PH hypothesis test.

**Supplementary Table 9** Multivariate PH assumption test.

**Supplementary Table 10** The 44 KEGG pathways enriched for differences between the high-risk and low-risk groups.

**Supplementary Table 11** List of miRNAs and lncRNAs that are regulatively associated with MET and AK4

# 2 Supplementary Methods

**S1. Data Download and Preprocessing**

TCGA-PAAD transcriptomic data were obtained from the UCSC Xena platform (https://xena.ucsc.edu/). Two expression matrices were downloaded: a count matrix standardized by log2(count+1) and a fragments per kilobase of transcript per million mapped reads (FPKM) matrix standardized by log2(FPKM+1). Differential expression analysis was performed using DESeq2 on raw count data, while all other downstream analyses utilized the FPKM matrix.

For GEO datasets (GSE28735, GPL6244; GSE62452, GPL6244; GSE57495, GPL15048; GSE79668, GPL11154), expression data were downloaded using the getGEO function with the parameter GSEMatrix = TRUE in R. Expression matrices were subsequently extracted using the exprs() function. These data were derived from pre-processed sequence matrix files provided by GEO, in which the original data contributors had performed background correction and normalization. Therefore, no additional robust multi-array average (RMA) normalization or background correction was applied in this study.

Uniform cleaning steps were applied to all datasets, including: (1) annotation of probe IDs to official gene symbols based on the corresponding platform annotation files (GPL6244, GPL15048, GPL11154); (2) removal of duplicate genes, retaining the probe with the highest average expression across all samples; (3) filtering of samples with missing values for survival time, survival status, or key clinical variables; and (4) removal of genes with missing expression values using the na.omit function in R.

**S2. Identification of T-RGs: Technical Details**

The ssGSEA algorithm was implemented using the R package GSVA (v 1.46.0) to compute enrichment scores for 28 immune cell types in TCGA-PAAD. Results were visualized as heatmaps using pheatmap (v 1.0.12). Differential immune cell abundance between the PC and control groups was assessed using the Wilcoxon test implemented in rstatix (v 0.7.2), with p < 0.05 as the significance threshold. Box plots were generated using ggplot2 (v 3.4.4).

Optimal ssGSEA score thresholds for each T cell type were determined using the surv_cutpoint function, and patients were stratified into high- and low-scoring groups accordingly. KM survival curves were constructed and compared between groups using the survminer package (v 0.4.9), with p < 0.05 considered statistically significant.

Sample quality control was performed using the GoodSamplesGenes function to detect and remove outlier samples. The optimal soft-threshold power was selected using the pickSoftThreshold function by evaluating the scale-free topological fit index (R²) and mean connectivity across a power range of 1–20; a power value yielding R² ≥ 0.90 and mean connectivity approaching zero was selected. Gene modules were identified using a dynamic tree-cutting algorithm with the following parameters: minimum module size of 300 genes (minModuleSize = 300) and deepSplit = 4 to enhance clustering sensitivity. Highly similar modules were merged using a height threshold of 0.25 (mergeCutHeight = 0.25). Module-trait correlations were computed between module eigengenes and T cell ssGSEA scores; modules with |cor| > 0.3 and p < 0.05 were designated as T-RG modules.

**S3. Identification of Candidate Genes and Functional Analysis: Technical Details**

Differentially expressed genes in GSE28735 (DEGs1) were identified using the limma package (v 3.54.0) with the threshold of |log2FC| > 0.5 and p < 0.05. Differentially expressed genes in TCGA-PAAD (DEGs2) were identified using DESeq2 (v 1.38.0)[20] with the same criteria. For both datasets, volcano plots were generated using ggplot2 (v 3.4.4) and heatmaps were visualized using ComplexHeatmap (v 2.14.0).

The upregulated genes of DEGs1 and DEGs2 were intersected, and the downregulated genes were intersected separately, using VennDiagram (v 1.7.3). The resulting upregulated and downregulated intersecting genes were combined to form DEGs3. The overlap among DEGs3, T-RGs, and G-RGs was subsequently determined using VennDiagram to obtain the final candidate gene list.

GO and KEGG enrichment analyses were performed using clusterProfiler (v 4.7.1.003), with p-adjust < 0.05 as the significance threshold. Results were visualized using ggpubr (v 0.6.0).

Functional gene interaction networks for the candidate genes were predicted using the GeneMANIA database (http://genemania.org), including co-expression, physical interaction, pathway co-membership, and shared protein domains.

**S4. Screening of Prognostic Genes: Technical Details**

Univariate Cox regression results were visualized using forestplot (v 3.1.1). PH assumption testing was performed using the survminer package (v 0.4.9). LASSO regression was implemented using glmnet (v 4.1-4) with lambda.min determined by 10-fold cross-validation. Expression differences in prognostic genes across GSE28735 and GSE62452 were assessed by the Wilcoxon test, and KM curves were generated using survminer (v 0.4.9).

**S5. Assessment and Verification of the Prognostic Model: Technical Details**

KM survival analysis was performed using the survival package (v 3.5-3). ROC curves and AUC values were calculated using survivalROC (v 1.0.3.1). Heatmaps were generated using pheatmap. The TCGA-PAAD-derived median cutoff (2.597068) was directly applied to GSE79668 for cross-cohort validation; dataset-specific medians were used for TCGA-PAAD (2.597068) and GSE57495 (9.40481) respectively.

**S6. Independent Prognostic Analysis: Technical Details**

Cox regression results were visualized using forestplot. The nomogram was constructed using the rms package (v 6.5.9). Calibration curves were generated using rms; slopes closer to 1.0 indicate higher predictive accuracy. DCA was performed using ggDCA (v 1.1). Risk score distributions across clinical subgroups were visualized as violin plots using ggplot2 (v 3.4.4).

**S7. GSEA: Technical Details**

Differential expression between risk groups was computed using DESeq2, with log2FC values ranked from largest to smallest as input for GSEA. The background gene set used was c2.cp.kegg.v2023.1.Hs.symbols.gmt obtained from MSigDB (https://www.gsea-msigdb.org/gsea/msigdb). GSEA was implemented using clusterProfiler, and results were visualized using enrichplot (v 1.20.3). The top five most significant pathways by p-value were selected for presentation.

**S8. Immune Microenvironment Analysis: Technical Details**

ESTIMATE scores were computed using the ESTIMATE algorithm (v 1.0.13). Immune checkpoint expression (48 checkpoints) and immune cell enrichment scores (28 cell types) were visualized using ggplot2 and pheatmap. Spearman's correlations were computed using the psych package (v 2.2.9). TMB and mutation profiles were analyzed using maftools (v 2.18.0).

**S9. Regulatory Network: Technical Details**

miRNA prediction was performed using the miRanda database (https://regendbase.org/tools/miranda) and miRDB (https://mirdb.org/) via the multiMiR package (v 1.24.0). Common miRNAs from both databases were identified by intersection using VennDiagram. Corresponding lncRNAs were predicted using starBase (http://starbase.sysu.edu.cn/). The lncRNA-miRNA-mRNA network was visualized using ggraph (v 2.2.1).

**S10. Drug Sensitivity: Technical Details**

IC50 values were predicted using the pRRophetic package (v 0.5) based on GDSC pharmacogenomic data (https://www.cancerrxgene.org/). Spearman's correlations between IC50 values and risk scores were computed using the psych package. The top 15 drugs ranked by p-value were visualized using ggplot2.

**S11. Immune Cell Infiltration Algorithms: Technical Details**

CIBERSORT estimates CD8⁺ T cell abundance via linear support vector regression-based deconvolution. EPIC applies constrained least squares regression with explicit non-negativity constraints. MCP-counter quantifies absolute CD8⁺ T cell abundance using normalized transcriptomic data. quanTIseq performs deconvolution-based quantification of CD8⁺ T cell infiltration. xCell derives CD8⁺ T cell enrichment scores using ssGSEA. All five algorithms were integrated using the R package immunedeconv, with the deconvolute function applied to transcriptomic expression matrices. CD8⁺ T cell estimates were extracted and correlated with GPR87 expression across three cohorts.

**S12. Detailed Experimental Protocols**

**S12.1 Cell Counting Kit-8 (CCK-8) assay**

Log-phase cells were seeded in 96-well plates (1,000 cells/well; performed in triplicate). At 24, 48, and 72 h, 10 μL of CCK-8 (Beyotime) was added and incubated for 2 h. OD450 was measured (BioTek) and analyzed using GraphPad.

**S12.2 5-Ethynyl-2’ -deoxyuridine (EdU) assay**

Cells were plated in 24-well plates, incubated with EdU (Beyotime) for 2 h, fixed with 4% paraformaldehyde for 15 min, permeabilized (Beyotime), and the nuclei were counterstained with Hoechst 33342. Three random fields per well were imaged, and proliferation was calculated as EdU-positive/total cells.

**S12.3 Transwell migration assays**

Cells were resuspended in serum-free medium (1 × 10⁵ cells/mL); 200 μL was added to the upper chamber, and 700 μL of medium with 20% FBS was added to the lower chamber. After 48 h, cells were fixed (4% paraformaldehyde), stained with 0.1% crystal violet, non-migrated cells were removed, and the migrated cells were counted in three random fields using an inverted microscope (Olympus IX73).

**S12.4 Colony-forming assay**

After transfection, 1,000 cells/well were seeded in 6-well plates and cultured for 2 weeks. The colonies were fixed, stained with 0.1% crystal violet, and counted.

**S12.5 Flow cytometry**

Control and siRNA-treated cells were stained with Annexin V–FITC (15 min at 4°C in the dark) followed by propidium iodide (Annexin V-APC/PI kit) and analyzed on a CytoFLEX flow cytometer.

**S12.6 IHC staining protocol**

Tissues were fixed in 4% paraformaldehyde, paraffin-embedded, and sectioned at 4 μm. Antigen retrieval was performed by heat-induced epitope retrieval. Sections were blocked with 3% BSA for 10 min. Primary antibodies were incubated overnight at 4°C, followed by species-specific secondary antibodies for 30 min. DAB chromogenic reaction was performed and sections were counterstained with hematoxylin, mounted with neutral balsam, and scanned using a panoramic scanner.

# 3 Full‑scan blots（Western Blot）
